# Deciphering the Code of Viral-Host Adaptation Through Maximum-Entropy Nucleotide Bias Models

**DOI:** 10.1093/molbev/msaf127

**Published:** 2025-06-03

**Authors:** Andrea Di Gioacchino, Ivan Lecce, Benjamin D Greenbaum, Rémi Monasson, Simona Cocco

**Affiliations:** CNRS UMR 8023, Laboratory of Physics of the Ecole Normale Supérieure and PSL Research, Sorbonne Université, Paris, France; CNRS UMR 8023, Laboratory of Physics of the Ecole Normale Supérieure and PSL Research, Sorbonne Université, Paris, France; Computational Oncology, Department of Epidemiology and Biostatistics, Memorial Sloan Kettering Cancer Center, New York, NY, USA; Physiology, Biophysics and Systems Biology, Weill Cornell Medicine, Weill Cornell Medical College, New York, NY, USA; CNRS UMR 8023, Laboratory of Physics of the Ecole Normale Supérieure and PSL Research, Sorbonne Université, Paris, France; CNRS UMR 8023, Laboratory of Physics of the Ecole Normale Supérieure and PSL Research, Sorbonne Université, Paris, France

**Keywords:** viral host adaptations, nucleotide usage, maximum entropy models, *Coronaviridae*, *Flaviviridae*, *Picornaviridae*, *Orthomyxoviridae*, human, avian, swine

## Abstract

How viruses evolve largely depends on their hosts. To quantitatively characterize this dependence, we introduce Maximum Entropy Nucleotide Bias models (MENB) learned from single, di- and tri-nucleotide usage of viral sequences that infect a given host. We first use MENB to classify the viral family and the host of a virus from its genome, among four families of ssRNA viruses and three hosts. We show that both the viral family and the host leave a fingerprint in nucleotide motif usages that MENB models decode. Benchmarking our approach against state-of-the-art methods based on deep neural networks shows that MENB is rapid, interpretable and robust. Our approach is able to predict, with good accuracy, both the viral family and the host from a whole genomic sequence or a portion of it. MENB models also display promising out of sample generalization ability on viral sequences of new host taxa or new viral families. Our approach is also capable of identifying, within the limitations imposed by the three-host setting, intermediate hosts for well-known pathogenic strains of Influenza A subtypes and Human Coronavirus and recombinations and reassortments on specific genomic regions. Finally, MENB models can be used to track the adaptation to the new host, to shed light on the more relevant selective pressures that acted on motif usage during this process and to design new sequences with altered nucleotide usage at fixed amino-acid content.

## Introduction

The recent COVID-19 pandemic has renewed the interest of the scientific community in zoonotic transmission of viruses (Parrish et al. 2008; Andersen et al. 2020) and in the subsequent evolutionary dynamics of viral adaptation to a new host. Several experimental (Starr et al. 2020; Moulana et al. 2022; Tenthorey et al. 2022) and computational (Rodriguez-Rivas et al. 2022; Tubiana et al. 2022) investigations have pointed out the effects of amino-acid mutations in the spike glycoprotein, in particular on the interaction with the human ACE2 receptor, which have conferred fitness advantages and resulted in selective sweeps of new variants (Kang et al. 2021; MacLean et al. 2021; Tan et al. 2022; Lee et al. 2025).

A related question is the identification of Pathogen-Associated Molecular Patterns (PAMPs) in a viral sequence (Akira and Hemmi 2003) and of their modifications as a result of adaption to the human environment and the need to alter innate immune recognition and response. This topic was previously explored for the H1N1 strain of the 1918 H1N1 influenza pandemic. In this context, it was shown that the viral genome evolved in a predictable way to lose CpG motifs (a cytosine followed by a guanine in the 5’-to-3’ sense) after entering the human host from an avian reservoir (Greenbaum et al. 2008, 2014). These computational analysis, together with the observation that most human-infecting viruses have low abundance of CpG motifs (Karlin et al. 1994; Shackelton et al. 2006; Di Giallonardo et al. 2017), were confirmed by the experimental identification of the CpG-dependent receptor specificity of the human Zinc-finger Antiviral Protein (ZAP, coded by ZC3HAV1 gene) (Gao et al. 2002; Takata et al. 2017; Ficarelli et al. 2020). Similar studies for the early evolution of SARS-CoV-2 have been carried out (Di Gioacchino et al. 2021; Kumar et al. 2022), showing a pressure to reduce CpG motifs in CpG-rich regions of the viral genome. More generally, understanding and controlling the innate immune response to foreign RNA is important for DNA and RNA vaccine design, in particular to avoid over-stimulating the host innate reaction (Zhang et al. 2023), while also optimizing for features, such as codon bias (Pardi et al. 2018).

The above issues are facets of the fundamental problem of determining how the interaction of a virus with its host is imprinted in the viral genome. Previous studies have shown that viruses in the same family accumulate mutations to use similar nucleotide patterns when they evolve in interaction with a specific host (Hall et al. 2013; Wille and Holmes 2020; Bloom et al. 2023). This idea has been in turn the cornerstone of a fruitful series of works to classify the host of a virus, hereafter intended as the organism it has been isolated from, based on its genome. Remarkably, methods that do not resort to sequence alignment perform, for this specific task, comparably well with alignment-based methods (Li and Sun 2018). These methods typically rely on the frequencies of *k*-mers (subsequences of length *k*) up to a given length $k_{\max}$, either alone (Tang et al. 2015; Brierley and Fowler 2021), together with other features such as physical–chemical properties of amino-acids (Young et al. 2020), or using a hybrid method that integrates alignment-based features (Babayan et al. 2018). Recently, deep neural networks were used to find the correct host of a given virus, completely bypassing the choice of the features used for a model (Mock et al. 2020). While most of these methods exhibit remarkable classification performance, there is a need for techniques effective at the classification task, while remaining simple to use, robust to generalization and interpretable. The latter point is particularly important for increasing our ability to detect zoonoses (Westgeest et al. 2014; Wille and Holmes 2020), for the molecular identification of PAMPs, which can indicate the possibility of aberrant innate activity in a host, and for the prediction of the evolutionary processes that a virus undergoes after switching host.

In this work, we address all these issues by taking a novel approach: we build a maximum entropy model, whose parameters are inferred to capture short-range (up to 3-mers) nucleotide usage patterns in viral genome sequences. This method is called Maximum-Entropy Nucleotide Bias (MENB) in the following.

Maximum-entropy models have been already used in various situations, including the modeling of protein sequences (Cocco et al. 2018; Mayer et al. 2022), of neural activity (Tavoni et al. 2017), and of social dynamics (Bialek et al. 2012), demonstrating the effectiveness and flexibility of this approach. In the context of viral evolution and identification of PAMPs in RNA sequences, the approach introduced here extends the classification based on motif usage, such as mononucleotide and dinucleotides (Lobo et al. 2009; Kapoor et al. 2010), and the selective force model previously introduced (Greenbaum et al. 2014; Tanne et al. 2015; Di Gioacchino et al. 2021) which reproduced the motif usage of individual nucleotides and for one 2-mer (CpG). In analogy to *k*-mer-based methods, ours does not require any alignment or annotation of the genetic sequence under analysis.

The paper is organized as follows. In Section “MENB: A Model for Host and Viral Family Classification”, we introduce MENB and show how it can be used to identify the host and the viral family in our setting limited to three hosts (human, avian, or swine) and four ssRNA viral families (*Coronaviridae*, *Flaviviridae*, *Picornaviridae*, *Orthomyxoviridae*). In Section “Comparison of MENB with VIrus Deep learning HOst Prediction (VIDHOP)”, we compare MENB with VIDHOP, a state-of-the-art method for host classification (Mock et al. 2020). In Section “Classification Performance and KL Distance between MENB Models,” we rely the decoding errors to the Kullback–Leibler (KL) divergence between the inferred models. We then benchmark MENB using for train or test data partial portions of the genome (Sections “Classification Performance on Partial Genomes” and “Classification performance on specific genomic portions reveals large genomic heterogeneities”).

In Sections “Classification Errors on Pandemic Strains Reveal Zoonoses and Previous Hosts” and “Classification Performance on Specific Genomic Regions Reveals Host-related Pressures and Recombinations or Reassortments,” we focus on *Coronaviridae* and *Orthomyxoviridae* to unveil the connection between host-prediction errors and zoonoses both at the whole-genomic scale and at the local scale of particular genomic regions. In Section “Viral Family and Host Decoding on Host and Viral Families not Included in the Dataset,” we investigate the generalization capability of MENB over new viral families and new hosts. In Section “Generative Power of MENB Models,” we study the generative properties of MENB to design new sequences with a desired bias in the nucleotide composition at fixed amino-acid content. In Section “Viruses Adapt to Their Host After Hosts Jumps: Applications to H1N1 Influenza and SARS-CoV2,” we use the inferred models to investigate modes of viral adaptation to new hosts for H1N1 Influenza A and SARS-CoV-2. Finally, in Section “MENB Model Parameters Reflect Biologically Relevant Features,” we describe the inferred selective force profiles and their changes after host-jumps and interpret them in terms of PAMPs and host-related pressures acting on nucleotide usage.

## Results

### MENB: A Model for Host and Viral Family Classification

Our unsupervised approach learns features associated with each *k*-mer up to k=3, and defines a probability distribution on viral sequences, in such a way that the frequencies of all *k*-mers sampled from this distribution match those observed in the training data. As shown in Methods Section “Maximum Entropy Justification,” this results in the following probability distribution for a viral sequence s:


$$p(s)\propto \exp\;\left(\sum_{a\in S}f_{a}^{\left(1\right)}n_{a}(s)+\sum_{ab\in S}f_{ab}^{\left(2\right)}n_{ab}(s)+\sum_{abc\in S}f_{abc}^{\left(3\right)}n_{abc}(s)\right),$$

S is the set of nucleotides, $n_{m}(s)$ is the number of times the *k*-mer motif m is present in s, and the parameters indicated by f are “selective forces” (Greenbaum et al. 2014) to be inferred from the training data. The inferred force parameters constrain the motifs usage in the model distributions and give a compressed representation of a single sequence or a set of sequences. Our approach extends previous works that considered CpG dinucleotide only, i.e. with $f_{m}=0$ except for m=CG (Greenbaum et al. 2014; Di Gioacchino et al. 2021).

To train our model, we collected viral sequences from the BV-BRC database (Olson et al. 2022), for three host classes: human, avian, and swine. A single sequence (of sufficient length) is in principle needed to train a model, as the number of force parameters is small (48 in total, see Methods Section “Gauge Choices for MENB Model”). However, as far as classification is concerned, we decided to train MENB on sets of 100 sequences to encompass in a single model sequences of different viral subfamilies or types. To have enough (≥50) remaining sequences to test predictions, we required the availability of at least 150 (different) viral genomes for each host class, this condition was satisfied for four viral families on which we have focused our study: *Coronaviridae*, *Flaviviridae*, *Picornaviridae*, and *Orthomyxoviridae* (focusing on Influenza A alone). The train and test dataset are precisely described in Methods Section “Data and Code Availability” and supplementary fig. S1, Supplementary Material online.

**Fig. 1.**
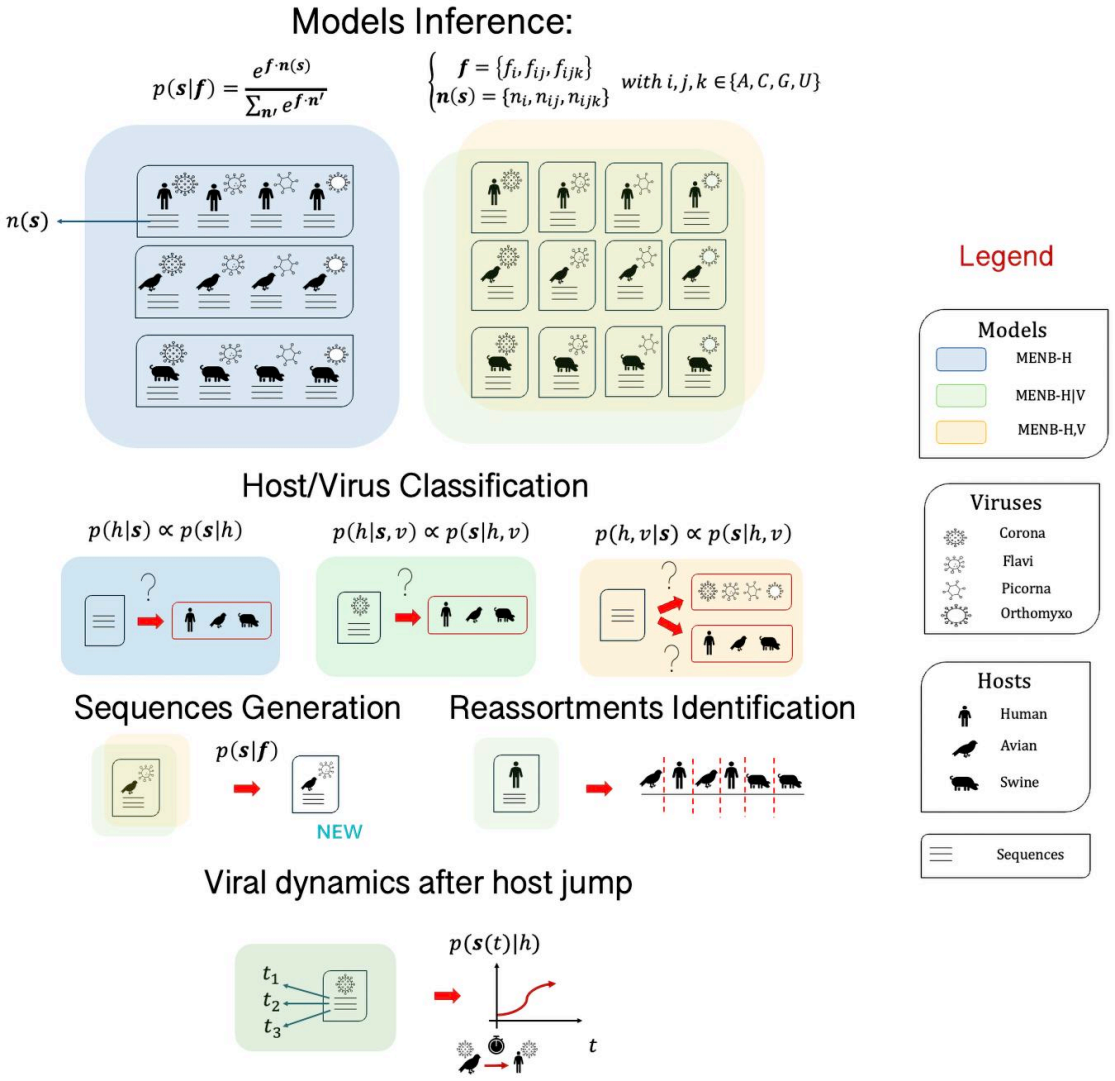
Pipeline. Maximum-Entropy Nucleotide Bias models (MENB) are trained on ssRNA sequences of four viral families: *Coronaviridae*, *Flaviviridae*, *Picornaviridae* and *Orthomyxoviridae*; and three hosts from which the viral genomes were isolated: Human, Avian, and Swine. In MENB models, the probability of a sequence, p(s|f), depends on its 1-2-3-mers usage, described by n(s), and the 1-2-3 force parameters f. The forces f are inferred from the n(s) of the sequences s in the training data. Three types of MENB models are introduced, differing by the data used for training and test: 12 MENB-H,V and 12 MENB-H|V models, one for each host and viral family, and 3 MENB-H models, one for each host independently from the viral family. Once trained, the models can predict the host from a test viral sequence through maximum-likelihood inference: MENB-H,V models predict both the host and the viral family from a sequence, MENB-H and MENB-H|V models predict the host only, the latter needing also information about the viral family. MENB models are also used to generate plausible sequences with altered *k*-mers biases, to identify previous hosts in specific influenza/COVID strains and segments/genomic portions due to intra-host reassortments/recombinations, and to study the adaptation dynamics of a viral strain after the jump to a new host.

We consider three strategies (graphically represented in Fig. 1).

- In the simplest one, called MENB-H, we group sequences according to their hosts *h*. All the sequences **s** belonging to the four viral families are pooled together and used to train a MENB model that approximates the probability p(s|h). Then, the use of Bayes formula p(h|s)∝p(s|h)  p(h), where p(h) is a prior distribution that we will consider uniform over the three hosts, allows us to predict the most likely host *h* for any test viral sequence.
- In a second strategy, called MENB-H|V, sequences are gathered according to their viral families *v* and their hosts *h*. For each family *v* and host *h*, we then learn a MENB model over the sequences s, with associated probability p(s|h,v). Using Bayes formula again, we obtain the joint posterior distribution p(h,v|s) for v,h given any sequence. If we assume that the viral origin $\left(v_{0}\right)$ of the sequence *s* is known, the most likely host can be inferred by ranking the three probabilities $p(h,v_{0}|s)$.
- In the third strategy, called MENB-H,V, we train the models as above but we assume that we ignore the viral family of the test sequences **s** from which we would like to infer the host. We can thus sum the probabilities p(s|h,v) defined above over the different viral families *v* (assumed to be *a priori* equally likely) to obtain the probability $p(h|s)\propto \sum_{v}p(s|h,v)$ that a virus is associated with a given host, and then infer the most likely host. However, for all viral genomes analyzed in this work, one out of the four families *v* contributes much more than the three others to the sum above. This dominating family is our guess for the viral family.

**Fig. 2.**
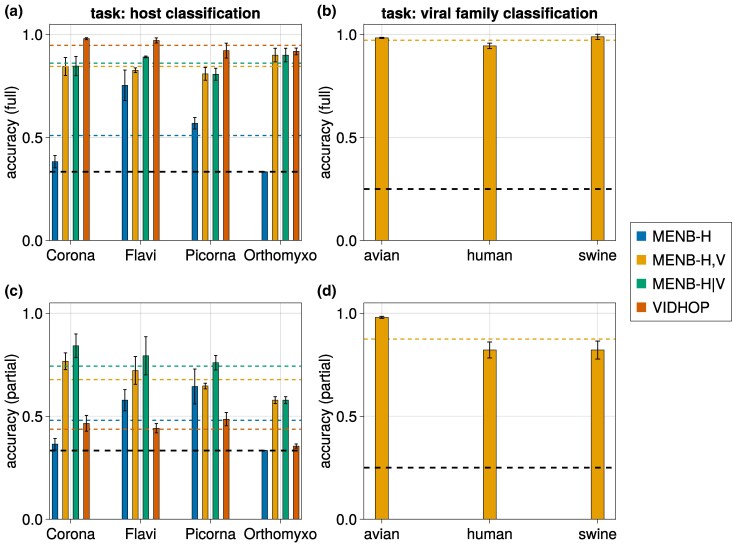
MENB models can predict host and viral family of viral genomes. a) Accuracy on the host classification task of MENB-H (blue bars), MENB-H,V (orange bars), and MENB-H|V (green bars) compared with VIDHOP algorithm (red bars) using, as MENB-H|V, only the correct viral family. The error bars are the standard deviations over the three partitions of the data in train and test sets (see supplementary fig. S1, Supplementary Material online). The horizontal dashed lines in color give the average accuracy for each model compared with the one of random guess (black). b) accuracy of MENB-H,V models in predicting the viral family on the test set. c) same as (a), but the training is done on the first half of the genome (for *Coronaviridae*, *Flaviviridae*, and *Picornaviridae*) or on all segments but PB2 (for *Orthomyxoviridae*), and the test is done on the remaining part of the sequence. See supplementary table S1, Supplementary Material online for the averages length of the genomes. d) same as (b), with the same task as described in (c).

The results of the host classification task on test viral sequences with the three strategies (MENB-H, MENB-H,V, and MENB-H|V) are displayed in Fig. 2a. We first notice that the viral agnostic models, MENB-H, has low accuracy, with an average over viral families of ∼51% (blue dashed line), only marginally better than random guessing (33%, black dashed line), due to performance comparable to random guessing for *Coronaviridae* and *Orthomyxoviridae*. These two viral families are the ones where zoonotic host jumps and genomic reassortement/recombination (Westgeest et al. 2014; Wille and Holmes 2020) have occurred and therefore we expect to have more difficulties in decoding the host, as it will be further discussed in Section “Classification Errors on Pandemic Strains Reveal Zoonoses and Previous Hosts.” The failure of this viral-agnostic host inference strategy shows that viral genomes are not free to evolve their nucleotide usage in a way that depends uniquely upon the host (Di Giallonardo et al. 2017). This result is not surprising as viral genomes are highly constrained by the proteins they code for (remarkably, the same genomic segment may locally code for multiple overlapping proteins through frameshift mechanisms) and by inter-protein interactions, e.g. for encapsulation.

It is thus reasonable to expect that family-dependent models (MENB-H,V) are more appropriate to capture these global constraints shared by the family. MENB-H,V gives an average performance in classification of 85% (yellow bars in Fig. 2a). The dominating family inferred with MENB-H,V corresponds to the true family with very high accuracy ≃97% (Fig. 2b). These results show that MENB-V,H is able to predict both the host and the family of a viral sequence. Performance of MENB-H|V for the host prediction task is similar to the one of MENB-H,V.

### Comparison of MENB with VIDHOP

We then compared our MENB models to other state-of-the-art approaches for host prediction. We considered VIDHOP (Mock et al. 2020), a deep-neural network designed specifically for this task, which can be obtained from a public code repository retrained by any user. While MENB can be used without information about the viral family of the target sequence, VIDHOP cannot generalize to viruses of different families, and was designed to work at fixed viral family (Mock et al. 2020). For the sake of comparison, we therefore focused on the strategy MENB-H|V.

We retrained VIDHOP and MENB on the same sequences, and compared their performance. Due to higher expressive power (number of learnable parameters) VIDHOP performance is better than the one of MENB (on average 93% accuracy (red) versus 86% accuracy for MENB-H|V (green)) in most cases and in particular for *Coronaviridae*, while being very similar for *Orthomyxoviridae*, as shown in Fig. 2a (green and red bars). However, VIDHOP requires many more resources (in terms of time and computational power) with respect to MENB (for instance, for each viral family VIDHOP requires ∼1 h on a 56-core CPU, while MENB requires <5 min on 3 cores).

We then wanted to confirm that our method, based on nucleotide motif usage only, could be applied to classify the host even with partial information about the viral sequence. As a particular challenging case, we aimed at predicting the host from a segment of the viral sequence not used in the training dataset. This task corresponds in machine learning to the ability to generalize to out-of-class samples, and is notoriously more difficult than in-class classification, where both train and test data are extracted from the same distribution. We expect that MENB can carry out this task if the *k*-mers statistics in the tested portion is similar to the one of the portion of the genomes used for training. To compare with VIDHOP we trained our model on the first half for *Coronaviridae*, *Flaviviridae*, and *Picornaviridae*, and on all segments but PB2 for *Orthomyxoviridae*, and used it to determine the host from the other parts of the viral genomes (we recall that the test sequences are distinct from the ones used for training). In this way, the classification is performed on sequences that are completely different (in terms of edit distance) from those used during training. As shown in Fig. 2c and d, MENB-H,V is able to identify quite precisely the viral family of the test part of the sequences (the average accuracy is ∼89%), and performs much better than a random classifier in determining the host (the average accuracy is ∼67%). The loss in performance with respect to the model trained from full sequences is moderate for all families, but more pronounced for *Orthomyxoviridae*. These results will be further discussed by an extensive benchmarking of MENB models on different genomic partitions in Section “Classification Performance on Specific Genomic Portions Reveals Large Genomic Heterogeneities.”

Remarkably, MENB performs sensibly better than VIDHOP, whose results are only marginally superior to random classification (Fig. 2c). This suggests that VIDHOP performance strongly relies on the large sequence similarities between training and test sequences, even if cross-validation during training is used to select the best model on a validation dataset.

### Classification Performance and KL Distance between MENB Models

**Fig. 3.**
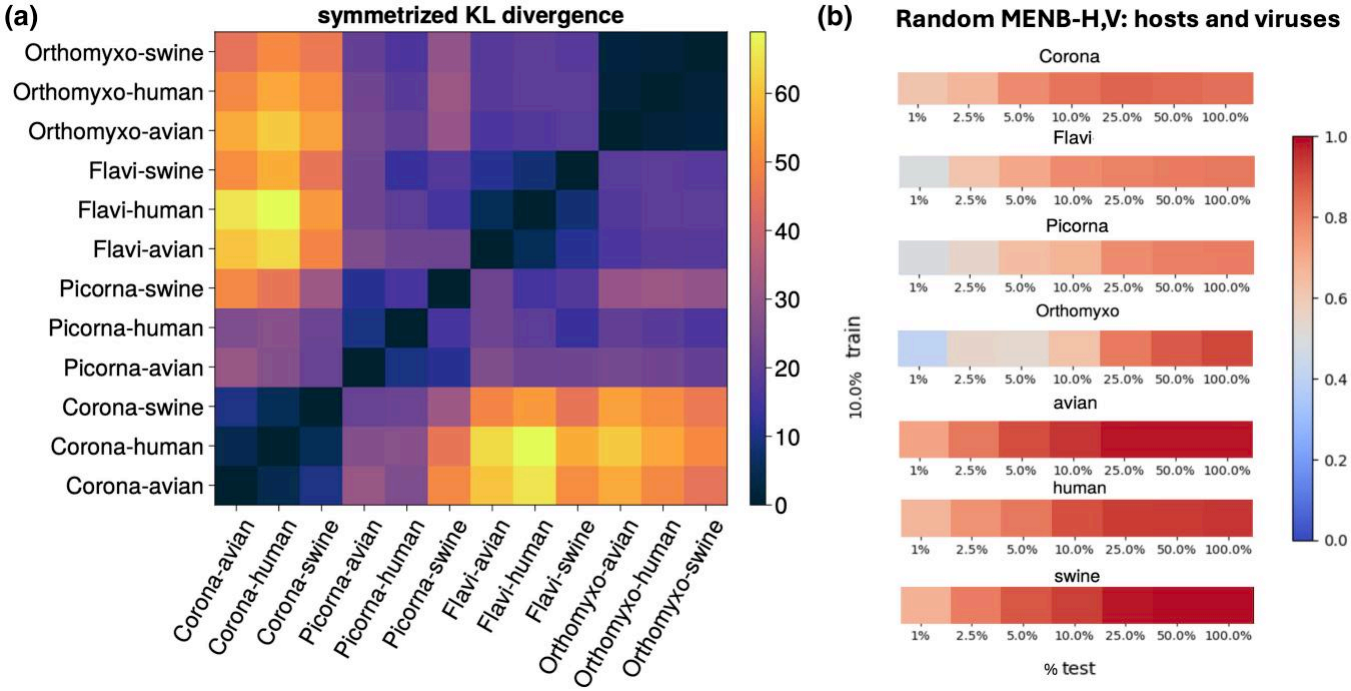
KL divergences between MENB models reflects robust accuracy in decoding a) Symmetrized KL divergences (color bar code) between all pairs of MENB H,V models. The divergence is computed from sequences having an arbitrary length of 1,000 nucleotides, see Methods Section “Computation of the Partition Function and Related Quantities.” b) Accuracy (color bar code) in host and viral family predictions with MENB-H,V models trained on segments, of 10% of the genomic length, randomly located along the genome, for randomly located test segments of the indicated percentages of the genomic length (see Methods Section “Data and Code Availability” for data partition and supplementary figs. S3 and S4, Supplementary Material online for results on additional lengths of segments in the train).

The classification performance of the MENB models is closely related to the differences between the probability distributions of the models associated with the various hosts and families. To precisely quantify such model dissimilarities, we use the symmetrized KL divergence (for a definition, see Methods Section “Computation of the Partition Function and Related Quantities”). The KL divergence between each pair of model distributions is given in Fig. 3a. Models trained on different viruses infecting the same host encode far more different probability distributions than models trained on viruses of the same family of different hosts, again suggesting that the use of nucleotides is more driven by virus-specific constraints than by host adaptation. This is compatible with the better performance of the MENB models in discriminating viral families rather than hosts, and the smaller divergences in the same viral family across hosts ultimately justify the choice of using MENB-H,V. We notice that *Orthomyxoviridae* viruses have smaller differences across hosts with respect to other viral families, probably due to host jumps and inter-host reassortments (Greenbaum et al. 2012; Westgeest et al. 2014; Wille and Holmes 2020), as will be further discussed in Section “Classification Errors on Pandemic Strains Reveal Zoonoses and Previous Hosts”.

### Classification Performance on Partial Genomes

In addition to the partitions used for comparison with VIDHOP, we have performed a systematic analysis of the effects of using partial genomes of different lengths to train and test our MENB models. We first consider genomic portions selected randomly along the train and test genomes. The segment lengths range from 1% of each genome up to full lengths (see Methods Section “Data and Code Availability” for more details and Fig. 11b for a graphical representation).

Prediction performance is almost independent from the length of the training portions, provided these are longer than the minimal length necessary to train the model, $150<L_{\mathrm{train}}^{\min}<400$ nucleotides depending on the viral family (see supplementary table S2, Supplementary Material online for minimal length specifications and supplementary fig. S3, Supplementary Material online). This result shows that a complete genome is not necessary for training, and that many fragments of different sequences which, on average, cover the entire genome give models of similar quality. The minimal length $L_{\mathrm{train}}^{\min}$ ensures that all motifs are observed at least once, which is a fundamental requirement to train our unregularized model. $L_{\mathrm{train}}^{\min}$ is of the order of $1/f_{\min}$, where $f_{\min}$ is the smallest 1,2,3-nucleotide motif frequency estimated from the set of viral sequences.

As shown in Fig. 3b, the length $L_{test}$ of the test segments above which the precision is stable is a fraction of the total genome of 5% to 10% for *Coronaviridae* and *Flaviviridae*, 25 % for *Picornaviridae*, which have the smallest genome, and 25% to 50% for *Orthomyxoviridae*. The deterioration of the accuracy with the smallest segments in *Orthomyxoviridae* is expected from the small KL divergence among different hosts (see Fig. 3a). The precision of the predictions as a function of the train and test lengths shows a similar trend for MENB-H|V and MENB-H,V (see supplementary figs. S3 and S4, Supplementary Material online top). For viral family predictions, as expected from the KL divergences, the accuracy reaches values >0.8 even when the models are tested on sequences as short as 2.5% of the genome length (see also supplementary fig. S4 (bottom), Supplementary Material online).

### Classification Performance on Specific Genomic Portions Reveals Large Genomic Heterogeneities

We have next performed genomic partitions corresponding to binary division of the genomes in 2, 4, 8, or 16 segments. Different settings for the train and test splitting were considered, in which these genomic portions are nonoverlapping or overlapping, and training is made with full genomes while test sequences are segments, or vice versa (see Methods Section“Data and Code Availability” and Fig. 11).

Genomic segmentation aims to further benchmark the host-decoding performance of the MENB models and to unveil the heterogeneities in genomic landscape. Such heterogeneities may be due local pressures to escape the host innate (e.g. on *CpG* abundance Ficarelli et al. 2020) or adaptive (e.g. on the Spike (S) glycoprotein in *Coronaviridae*, or the hemagglutinin (HA) and neuraminidase (NA) for *Orthomyxoviridae* (Jenkins and Holmes 2003; MacLean et al. 2021; Tan et al. 2022)) immune systems, or come from intra-host recombinations and reassortments (Westgeest et al. 2014).

**Fig. 4.**
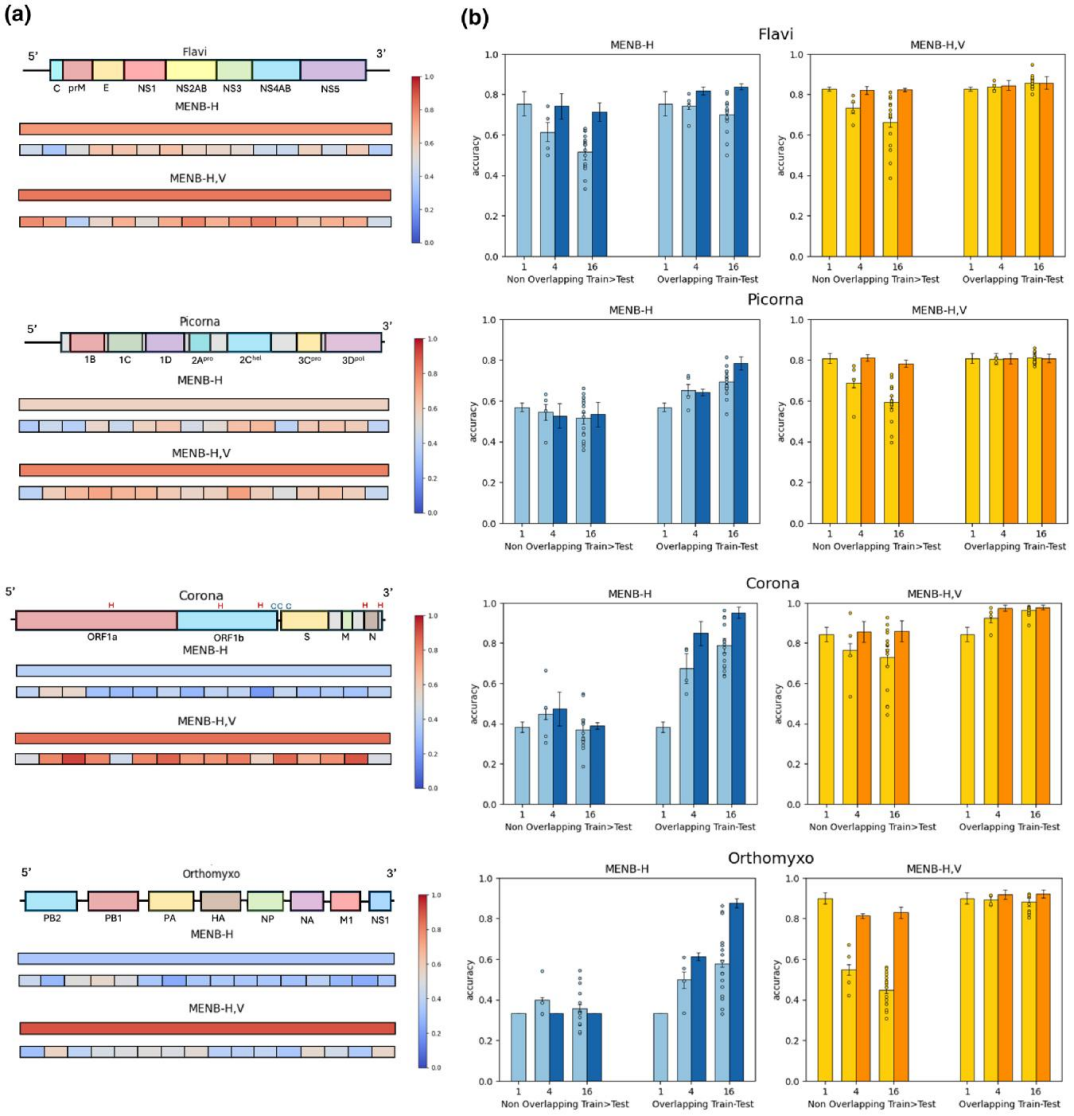
Performance of MENB models for host predictions on genomic segments. a) MENB-H and MENB-H,V accuracy (color-bar code) for host predictions from full genomes and partitions in 16 segments in Nonoverlapping Train>Test (the train and test regions are complementary genomic portions, see Methods Section “Data and Code Availability”). For *Coronaviridae*, H indicate reassortments hotspots and C coldspots. b) Bar plots of MENB-H and MENB-H,V host predictions accuracy for full genome compared with the 4-, 16-fold segmentation for nonoverlapping and overlapping setting. Lighter bar plots show the average over segments and its standard deviation (over three partitions of the data in train and test) while single-segment values are shown with dots. Darker bar plots give MaxSum accuracies with standard deviations. In the figure gene segmentation of labelled regions taken from, respectively (Zhao et al. 2021; Zell et al. 2017; Di Gioacchino et al. 2021; Carascal et al. 2022) and hot/coldspots from Lytras et al. (2022).

As shown in Fig. 4a (by the color scale) and Fig. 4b (by the dots in the bar plots), we observe, for all viral families, a large segment-to-segment dispersion of the prediction performance for the MENB-H model and for the nonoverlapping train-test settings of MENB-H,V (see supplementary figs. S5 to S7, Supplementary Material online for intermediate partitions). To combine information from single segments we decode the host as the one having maximal sum of probabilities over segments (procedure MaxSum, see Methods Section “MaxSum Inference Method”). As shown in Fig. 4b (darker bar plots), MaxSum not only allows us to recover full-genome classification accuracy, but also provides improvement in the cases of MENB-H models learned on overlapping segments and in the case of MENB-H,V on overlapping segments with *Coronaviridae*.

We next discuss the decoding heterogeneities by viral family. In *Flaviviridae*, vector-borne virus, a reduced positive selection from the host adaptive immune system has been documented (Worobey et al. 1999; Woelk and Holmes 2002; Jenkins and Holmes 2003) and no intra-host recombination has been observed. When decreasing the segment size of the tested nonoverlapping genomic portions, the mean accuracies deteriorates as statistically expected. Badly decoded segments in the 16-fold fragmentation include the last fragments corresponding to long 3’ UTR (Fig. 4b); not surprisingly, the model trained on coding region including translational constraints is not optimal for noncoding regions.

*Picornaviridae* are principally transmitted by fecal–oral route, local positive pressures to escape the adaptive immune system of the host are expected. Intra-host recombinations of the viral genome are very frequent, but not inter-host ones (Kempf et al. 2019). Since *Picornaviridae* have small genomes, the loss of performance in the nonoverlapping setting due to the 5’ and 3’ UTR is particularly clear for the first and last segments in the 16-fold segmentation (Fig. 4a), while the extremities have large accuracy in the overlapping setting (see supplementary fig. S7, Supplementary Material online). Some genomic portions carry a better host fingerprint, as they allow for better decoding the host compared to the entire genome, presumably due to positive pressures to adapt and escape the host.

*Coronaviridae* are linear viruses where both local pressures due to innate/adaptive host systems are expected, and intra-host jumps and recombinations take place (Lytras et al. 2022). This complex genomic landscape (Di Gioacchino et al. 2021; MacLean et al. 2021) is reflected in large fluctuations in accuracy in the nonoverlapping setting both for MENB-H and MENB-H,V models (Fig. 4a,b). Segments containing a better host fingerprint are in particular the one containing ORFS coding for the Spike protein, under strong pressure to escape the host (Dhama et al. 2020; MacLean et al. 2021; Lytras et al. 2022; Tan et al. 2022). On the contrary, performance decreases on the initial and final segments in the 16-fold segmentation, containing non coding regions and a part of ORF1a at the beginning and ORF10 and NORF at the end. As reported in Fig. 4, there is some correspondence between recombination coldspots/hotspots and segments with good and bad accuracy. Well-characterized recombination hotspots are in particular located in ORF1ab and the 3’ extremity containing ORF10, and ORFN (Lytras et al. 2022). The portion containing the ORF1a hot spot is less accurately decoded also in the overlapping setting (supplementary fig. S7, Supplementary Material online). Large CpG forces with respect to the rest of the genome in the human strains SARS-CoV-2, MERS, SARS have already been identified at the extremities of the *Coronaviridae* genome and in particular ORFN (Ficarelli et al. 2020; Velazquez-Salinas et al. 2020; Di Gioacchino et al. 2021; MacLean et al. 2021).

*Orthomyxoviridae* have fragmented-genomes, which undergo inter-host reassortment (Smith et al. 2009; Wong et al. 2010). As the genome is fragmented, the UTRs are found on each segment. The overall performance is low with MENB-H models and largely decreases when decoding with MENB-H,V models from nonoverlapping partial portions of the genome even for partitions in two halves (as already noticed in Fig. 2). Some segments, however, carry a better host fingerprint; in particular the ones in MENB-H,V containing PB2, NA, and NS (Fig. 4a).

A finer analysis of particular genomic regions for *Coronaviridae* and *Orthomyxoviridae* will be carried out in Section ‘Classification Performance on Specific Genomic Regions Reveals Host-related Pressures and Recombinations or Reassortments.”

MENB-H|V displays performance very similar to MENB-H,V on segmented genomes (supplementary figs. S6 and S7, Supplementary Material online). Lastly, as shown in supplementary fig. S8, Supplementary Material online, the decoding of the viral family with MENB-H,V is generally very accurate, for all hosts, even when decreasing the size of segments; the accuracy in the overlapping setting, as well as with MaxSum is very large (>85%).

### Classification Errors on Pandemic Strains Reveal Zoonoses and Previous Hosts

**Fig. 5.**
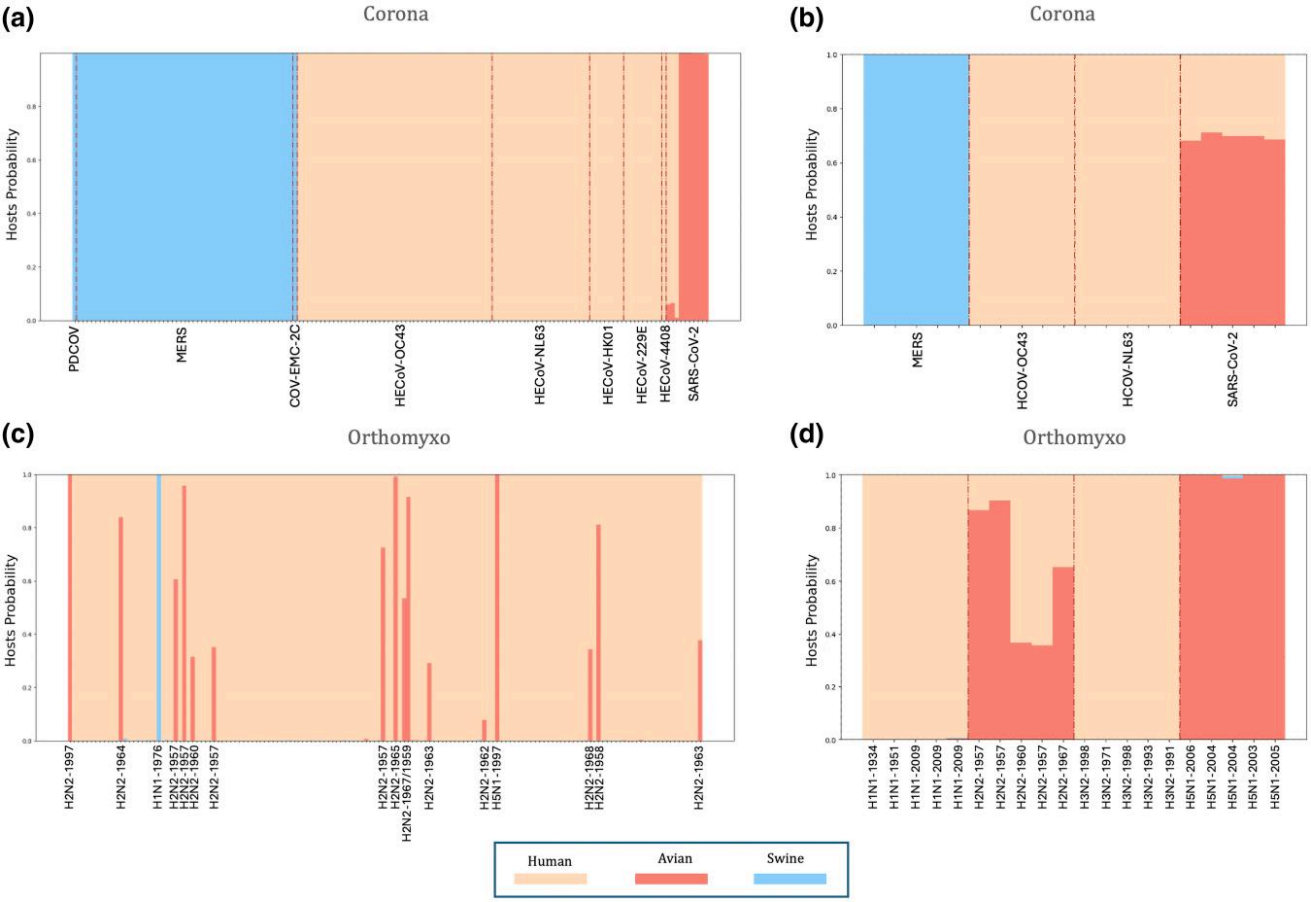
MENB-H|V Host probability for human *Coronaviridae* and *Orthomyxoviridae* human test sequences: decoding errors and zoonoses. a) MENB-H|V host probabilities of the 150 Human Corona here ordered by strains. The human host for all sequences are correctly predicted apart for MERS, of camel origin, and sequences of PDCoV of known swine origin and CoV-EMC-2C phylogenetically related to bat CoVs (Cotten et al. 2013). c) Host probability on the 150 test sequences in human *Orthomyxoviridae*. Decoding errors come from outbreaks after jumps from avian flu (H2N2 and H5N1 strains) and swine flu (H1N1 strain). b and d) Hosts-probabilities (averaged over the MENB-H|V models built on the 3 partitions of the standard train sets of supplementary fig. S1, Supplementary Material online) for two specific datasets of 20 full sequences for *Coronaviridae* and *Orthomyxoviridae* families, see supplementary fig. S2a, Supplementary Material online.

We have dug on the decoding performance of MENB-H|V for *Coronaviridae* and *Orthomyxoviridae* viral families where host jumps have caused several pandemics. In *Coronaviridae*, the MENB-H|V model (Fig. 2a) on the whole genome and standard test datasets (supplementary fig. S1, Supplementary Material online) has an average accuracy of 84.7%: avian are recognized at 100%, swine at 94% and human at 60%. The MENB-H|V host probability is plotted in Fig. 5a for the 150 human sequences in the test set labeled by their species. Sixty sequences give nonhuman host: 51 belong to MERS, a highly pathogenic virus, transmitted to humans from infected dromedary camels (Omrani and Shalhoub 2015; Brierley and Fowler 2021). Such sequences are predicted to be associated with the host “swine” in our three-host setting, in agreement with the classification of camelid sequences as swine, which we will discuss in Section “Viral Family and Host Decoding on Host and Viral Families not Included in the Dataset” and in supplementary fig. S10, Supplementary Material online. Other mismatches are a Porcine Deltacorona, of swine origins, identified as swine and a human betacoronavirus 2c EMC/2012 from Middle East, phylogenetically related to bat CoVs (Cotten et al. 2013), which are also identified as swine (see supplementary fig. S10, Supplementary Material online and Section “Viral Family and Host Decoding on Host and Viral Families not Included in the Dataset”). Furthermore, three SARS-CoV-2 sequences are identified as human (with nonzero avian host probability) and seven as avian, in agreement with a recent jump to the human host.

We have tested the MENB-H|V models on a specific set of five sequences for each human *Coronaviridae* strains of MERS, HCoV-OC43, HCoV-NL63, and SARS-CoV-2, confirming the above picture, as shown in Fig. 5b. Our results agree with previous analysis focused on elucidating the host jumps of *Coronaviridae* (Brierley and Fowler 2021) and decoding of MERS as camelid. Similarly, among the 150 human *Orthomyxoviridae* sequences in the test set, as shown in Fig. 5c, 11 are decoded as nonhuman: 8 from the H2N2 1957 to 1958 Asian-flu pandemic have nonzero probabilities to belong to avian and human hosts, 1 sequence decoded as swine host is from the 1976 swine flu outbreak and 2 sequences decoded as avian are from the H5N1 avian flu outbreak. We have further built a test set from five sequences for each influenza subtypes H1N1, H2N2, H3N2, H5N1 (supplementary fig. S2a, Supplementary Material online). As shown in Fig. 5d the five sequences from the H2N2 Asian-flu 1957 to 1958 pandemic have large probabilities of being from avian and human hosts, according to MENB-H|V models, and all the recent H5N2 sequences are decoded as avian.

Overall our findings confirm that for highly pathogenic variants which are not yet adapted to the human host, both in *Coronaviridae* and *Orthomyxoviridae*, the MENB-H|V models correctly decode, within the limitations imposed by the three-host setting, the previous host from which the virus has jumped into human, not the host from which the sequence has been sampled.

### Classification Performance on Specific Genomic Regions Reveals Host Related Pressures and Recombinations or Reassortments

We have then sharpened the genomic partitions of Section “Classification Performance on Specific Genomic Portions Reveals Large Genomic Heterogeneities,” for specific coding and noncoding regions in *Coronaviridae* and *Orthomyxoviridae* to investigate if MENB-H|V models detected region-dependent pressures to escape the host immune system and intra-host recombinations or reassortments in documented viral outbreaks.

**Fig. 6.**
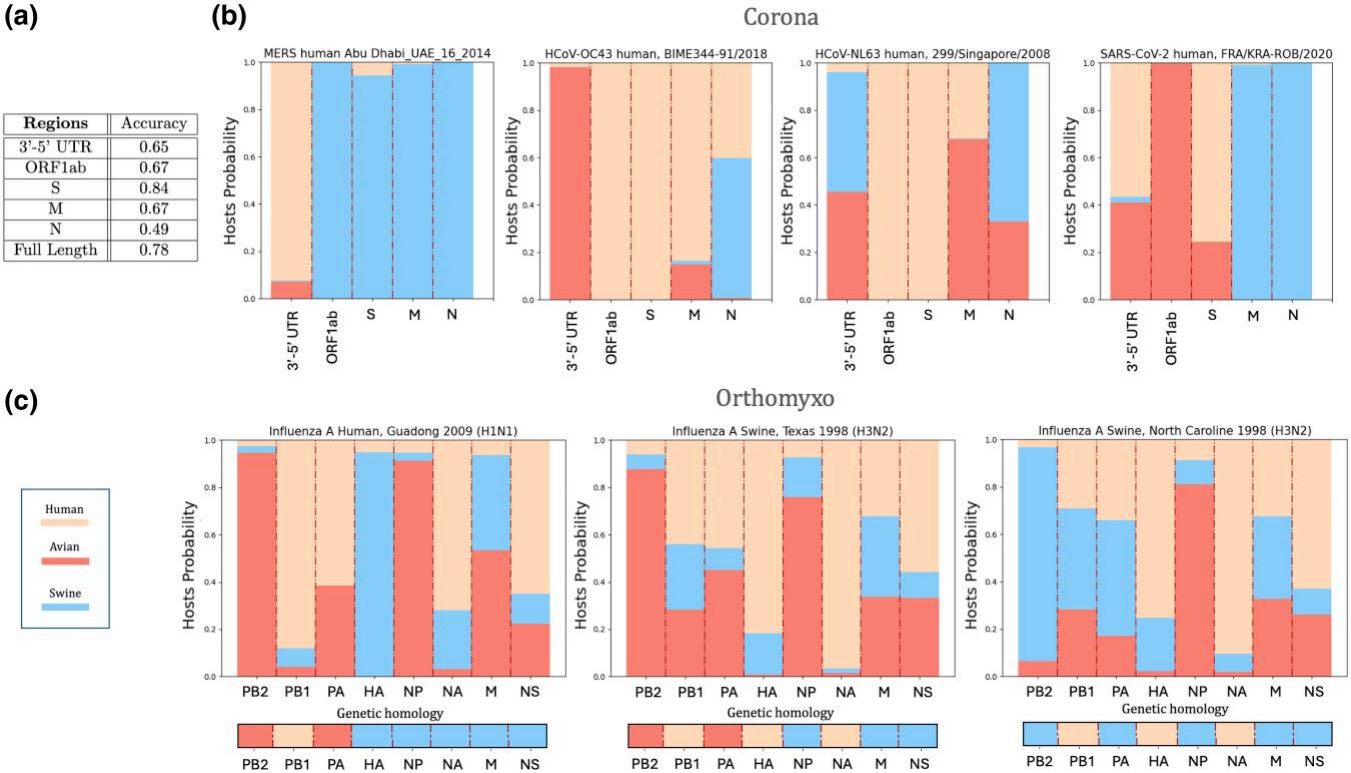
Host predictions for *Coronaviridae* and *Orthomyxoviridae* genomic segments: decoding errors are related to recombinations or reassortments after host jumps. a) Average accuracy of MENB H|V on 3’ to 5’ UTR, ORF1ab, ORFS, ORFM, and ORFN of 90 sequences of *Coronaviridae* (30 avian IBV, 30 Human SARS-CoV-2, and 30 Swine PDCoV). b) Average host probabilities decoded from 3’ to 5’ UTR, ORF1ab, ORFS, ORFM, and ORFN on four human *Coronaviridae* sequences of MERS, HCoV-OC43, HCoV-NL63 and SARS-CoV-2 (similar results on four additional sequences for each clade are shown in supplementary fig. S12, Supplementary Material online). c) Average host probabilities from the segments of three influenza A sequences: a human sequence from the H1N1 outbreak of 2009, with triple reassortment predicted by phylogenetic analysis (bottom), a sequence from swine influenza A H3N2, with triple reassortment predicted by phylogenetic analysis (bottom) and a sequence from swine influenza A H3N2, with double reassortment as predicted by phylogenetic analysis (similar results on 20 additional H1N1, H2N2, H3N2, and H5N1 sequences are shown in supplementary fig. S13, Supplementary Material online). The averages are computed over the MENB-H|V models built on the three partitions of the standard train sets of supplementary fig. S1, Supplementary Material online.

Figure 6a shows the average performance of MENB-H|V models (trained on the main dataset), when tested on specific genomic regions (5’ to 3’ UTR, ORF1ab, and the structural ORFS, ORFM, and ORFN) for a new test set of 90 *Coronaviridae* made of 30 sequences for each host (supplementary fig. S2b, Supplementary Material online). The ORFS has the largest accuracy, 84%, in agreement with the large immune pressure on this region (Tan et al. 2022), followed by ORF1ab, ORFM, and noncoding 5’ to 3’ UTR (65% accuracy), while ORFN is the region with lowest decoding performance (49% accuracy). When decoding the hosts with a MENB-H,V model trained on the same restricted dataset of 90 sequences, we obtain similar results, both in the full-genome and nonoverlapping settings. In particular, ORFS and ORF1ab are the best-decoded regions and ORFN the worst one, see supplementary tables S3 and S4, Supplementary Material online.

Figure 6b shows the decoding probability for the specific genomic regions in four human *Coronaviridae* of MERS, HCoV-OC43, HCoV-NL63, and SARS-CoV-2 strains, belonging to the test set used in Fig. 5b. As shown in supplementary fig. S12, Supplementary Material online, the other four sequences for each strain behave similarly. While the full genome analysis in Fig. 5b miss-classifies the host of MERS and SARS-CoV-2 as, respectively, swine and avian, segment analysis reveals a finer pattern, in overall agreement with the segment-dependent performance reported in Fig. 6a: MERS are predicted as swine for all ORFS, but correctly predicted as human on noncoding 5’ to 3’ UTR, in agreement with Brierley and Fowler (2021); SARS-CoV-2 is classified as human (with nonzero avian probability) in ORFS and in 5’ to 3’ UTR, avian in ORF1ab, and swine in ORFM and ORFN, unveiling a faster adaptation to the human host in ORFS (Guan et al. 2003; Dhama et al. 2020). For the two HCoV sequences, ORF1ab and ORFS are predicted as human, while ORFN is not, and ORFM is predicted as human in HCoV-OC43 and avian in HCoV-NL63.

Finally, we focus on the influenza segments for one sequence of the human 2009 H1N1 outbreak, and two swine sequences of the H3N2 outbreak that were previously characterized. As shown in Fig. 6c our model correctly predicts the triple reassortment on the sequence from the human H1N1 2009 outbreak (Shinde et al. 2009; Smith et al. 2009). According to the model, the PB2, PB1, and HA have for hosts, respectively, avian, human and swine. For the sequence of the swine H3N2 1998 outbreak (Zhou et al. 1999) characterized by a triple reassortment, MENB-H|V correctly predicts the human origins of PB1, HA, NA, and the avian origin of PB2. MENB-H|V finally correctly predicts the double reassortment in a 1998 swine H3N2 (Zhou et al. 1999) strain with HA, NA from humans, PB2 and PA from swine. More uncertain predictions are given for the other segments and the NP segment is always predicted as avian. To summarize, all large confidence predictions agree with genetic homology and the reassortments detected by phylogenetic, except for NP.

The HA, NA, PB2, and NP segments analysis for the test set of 20 *Orthomyxoviridae* human sequences in H1N1, H2N2, H3N2, and H5N1 flu outbreaks of Fig. 5, shows similar reassortment patterns (see supplementary fig. S13, Supplementary Material online) in particular the triple reassortments for the H1N1 2009, and the H5N1 2004 to 2006, and the H3N2 swine origin.

As a general finding ORFS in human *Coronaviridae* and NA in *Orthomyxoviridae* best decode the human host, as expected from the larger positive pressure to adapt to the human host, while ORFN coding for the nuclear capsid protein in *Coronaviridae* and the nucleoprotein NP in *Orthomyxoviridae* are often decoded as nonhuman. These results are compatible with the previous observation of ORFN being an hotspot of recombination (Lytras et al. 2022) and atypical in CpG content with respect to the rest of the genome (Ficarelli et al. 2020; Di Gioacchino et al. 2021); they also agree with the avian like origin of NP identified by phylogenetic studies (Gammelin et al. 1990; Surjit and Lal 2008).

### Viral Family and Host Decoding on Host and Viral Families not Included in the Dataset

To assess the generalization capability of MENB-H,V, we have benchmarked our model on new host and viral families.

We have first studied how MENB-H,V decodes the viral family from sequences of unknown hosts (Brierley and Fowler 2021) but known viral families. We have considered sequences from six new hosts for *Coronaviridae* and three new hosts for each other viral family (see supplementary fig. S2d, Supplementary Material online for detailed information on the dataset composition). For each new host, we have taken five test sequences and used MENB-H,V (trained on the main dataset) to decode the viral family and the host. The viral family is classified with 100% accuracy. Decoded hosts are shown in supplementary fig. S10, Supplementary Material online. Interestingly, for *Coronaviridae*, canine, bovine, feline, and rodent are decoded as human. This large variety of hosts assigned to human may be due to phylogenetic proximity but also to zoonotic transmissions (Dhama et al. 2020). Moreover, each bat sequence is assigned to one of the three hosts, which is compatible with bats acting as viral reservoirs. Camels are decoded as swine, confirming the decoding of MERS host as swine in Fig. 5.

Compatibly with reassortment, multiple possible hosts have nonzero probabilities on the *Orthomyxoviridae* sequences coming from bovine, canine, and feline hosts. The correct viral family is also decoded for bovine sequences of influenza D genus which is different from the training set containing only influenza A genus. Moreover, the bovine influenza D sequence is decoded as swine and was originally isolated from a piglet (Sreenivasan et al. 2019).

Secondly, we have tested MENB-H,V on sequences belonging to different viral families (see supplementary fig. S2c, Supplementary Material online for dataset composition and supplementary fig. S11a, Supplementary Material online for results). To this aim, we have collected 30 virus sequences in the *Caliciviridae* family, from avian, human and swine hosts. Calicivirus are positive single stranded RNA viruses of the order *Picornavirales*. The decoded viral family is *Picornaviridae* in 86.7% of cases, and the host is well decoded in 50% of cases. A comparable host accuracy of 48.8% (above the random prediction of 0.33) is obtained for an additional set of 90 sequences belonging to single stranded RNA viral families more taxonomically distant to the ones in the train set (supplementary fig. S11b, Supplementary Material online): *Paramyxoviridae*, *Rhabdoviridae*, and *Arteriviridae*. For the latter dataset, the decoded viral families are therefore difficult to interpret. Interestingly, in the above dataset, MENB-H is less accurate for host predictions (33% for Calicivirus and 42% for the other families), revealing that the MENB-H,V model is able to capture some homology and similar constraints in nucleotide usage even among distant viral families.

### Generative Power of MENB Models

**Fig. 7.**
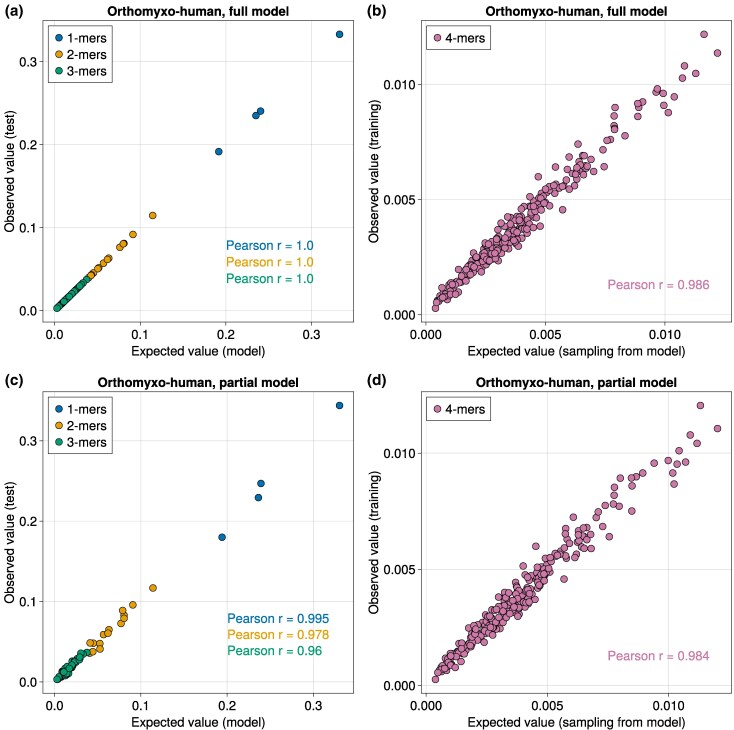
MENB models generalize well to test sequences and higher-order motifs. a) Frequency of nucleotides, 2-mers and 3-mers (colors in legend) observed in the test set of full human *Orthomyxoviridae* sequences versus the value obtained analytically from the inferred MENB model. b) Same as a for the MENB model trained on human *Orthomyxoviridae* sequences without the segment coding for PB2. c) Frequency of 4-mers observed in the training set of full human *Orthomyxoviridae* sequences versus the value obtained from sequences sampled from the inferred MENB model. d) Same as (c) for the MENB model trained on human *Orthomyxoviridae* sequences without the segment coding for PB2.

We tested the generative power of MENB, focusing first on *Orthomyxoviridae* sequences. The MENB model reproduces, as expected, the 1-, 2-, and 3-mer statistics of the training set (supplementary fig. S18, Supplementary Material online). The agreement between the statistics of *k*-mers predicted by the model and the ones computed from sequences also holds for test data, not used for training (Fig. 7a). Performance slightly worsen when training is done from a portion of the genome only, and the test set contains new sequences with unseen parts of the genome (Fig. 7c), further showing how nucleotide usage biases encompass the full viral sequences and can be learned from partial knowledge of the viral genomes.

We next investigated how well MENB could reproduce higher order statistics of *k*-mers, beyond the values k=1,2,3 considered for training. To do so, we sampled sequences from the probability distribution encoded by MENB models (using a standard Metropolis–Hastings algorithm) and compared the 4-mer frequencies of these sampled sequences with those of the training dataset. In Fig. 7b,d, we show that MENB model almost perfectly capture the 4-mer statistics.

**Fig. 8.**
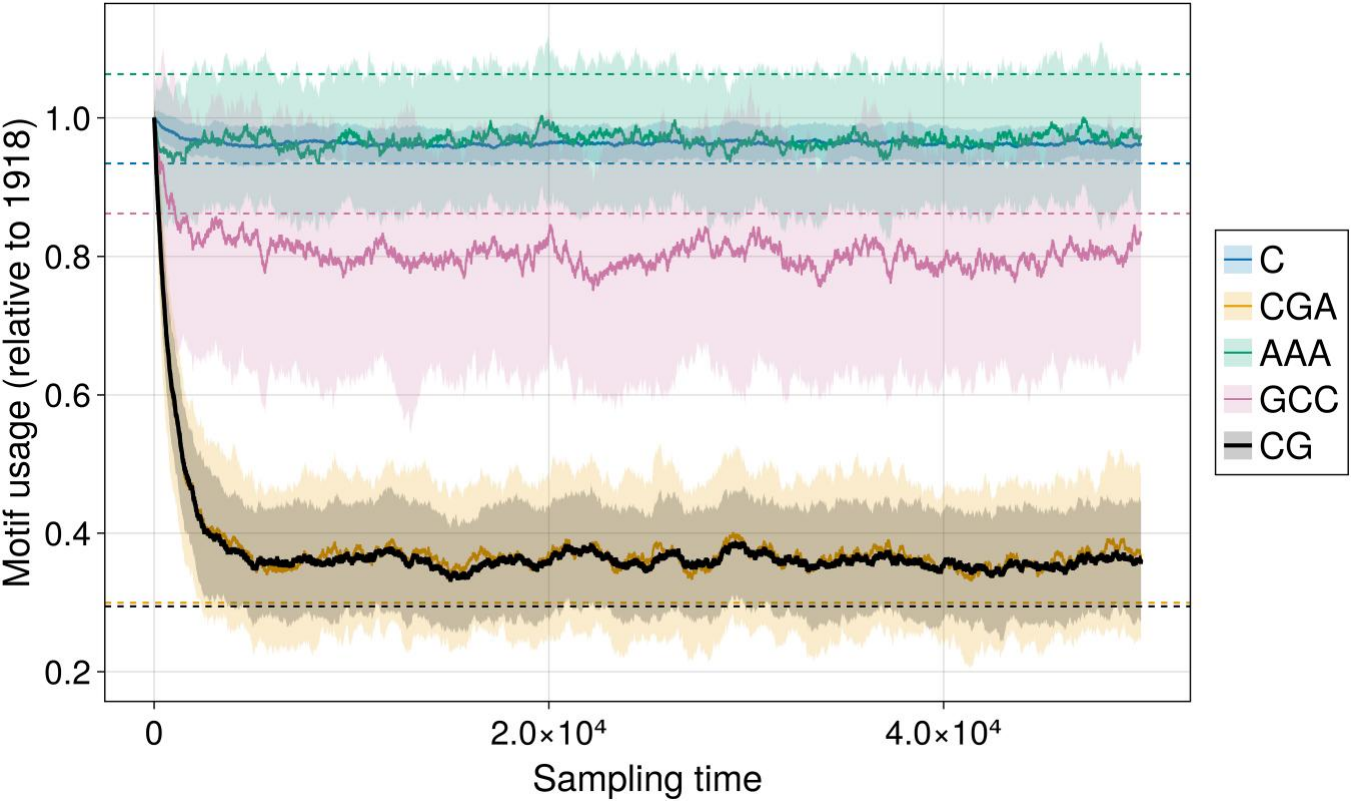
MENB models can be used to design new sequences coding for the same proteins and with different nucleotide usage. Motif usage (relative to the original sequence) during synthetic evolution of the PB2 coding sequence from 1918 H1N1 strain under a MENB-H|V model enforcing lower usage of CpG dinucleotides (black). Other representative motifs containing CpG or not are shown by different colors (in legend), see supplementary fig. S21, Supplementary Material online for all the motifs. Sampling time is in Metropolis–Hasting sampling steps. Solid lines were obtained as averages of 100 independent evolutions; shaded areas denote one standard deviation. Dashed lines denote the expected motif usage releasing the constraint to keep the amino-acid content.

In Fig. 8, we further show how we can leverage MENB models to change the nucleotide usage of a protein-coding sequence, while keeping the amino acids fixed. Increasing CpG content of HIV sequences at fixed amino-acid content was important to experimentally demonstrate that the ZAP protein targets CpG-rich sequences (Takata et al. 2017; Ficarelli et al. 2020). As an illustration, we considered the PB2 coding region of the 1918 H1N1 strain and wanted to reduce its number of PAMP-associated CpG motifs (Greenbaum et al. 2008, 2014). We synthetically evolved the 1918 sequence through Metropolis–Hasting sampling of the sequence distribution p(s) (see Section “Metropolis–Hasting Algorithm to Evolve Sequences”) using the force parameters inferred from the same sequence, apart from $f_{CG}$ that we fixed to $f_{CG}=-1.9$. Such $f_{CG}$ value is close to the average value in the human genome (Greenbaum et al. 2014) and is sensibly lower than the one in the original H1N1 strain ($f_{CG}=-0.6$). To leave the original amino-acid content of the 1918 sequence unchanged, only synonymous mutations in the Metropolis–Hasting sampling dynamics were accepted (Chatenay et al. 2017). As shown in Fig. 8, the changes in the nucleotide content mostly affected CpG dinucleotides and CpG-containing 3-mers, while other dimers and trimers were generally conserved, see supplementary fig. S21, Supplementary Material online. Generation of synthetic sequences under fixed constraint on other motifs could be analogously carried on by changing the corresponding forces.

### Viruses Adapt to Their Host after Hosts Jumps: Applications to H1N1 Influenza and SARS-CoV2

We demonstrated that our model can infer, from their nucleotide statistics alone, the host of viral sequences and detect the previous host in zoonoses. Here we show how the model describes the evolution of a viral strain to the new host after a host jump. We focus on the case of 1918 H1N1 influenza pandemics: we collected all PB2 segments available in our dataset associated with the H1N1 strain up to 2008.

**Fig. 9.**
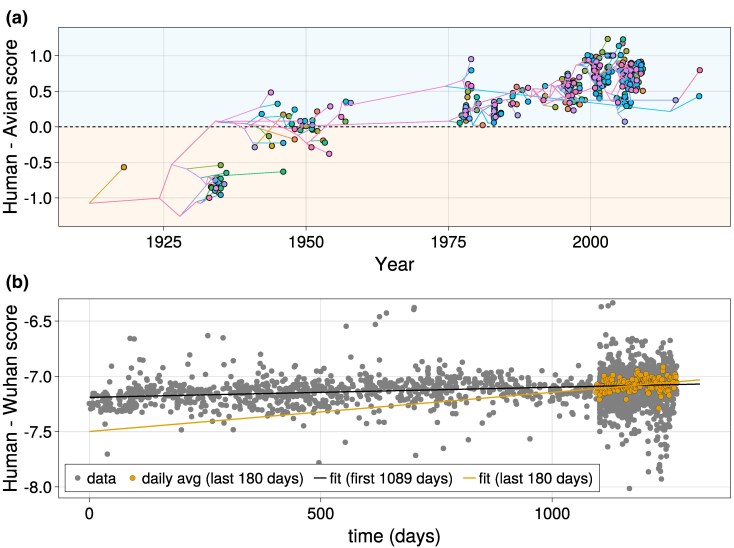
MENB models can be used to quantify host adaptation dynamics after host jumps. a) Log-likelihood difference of the MENB *Orthomyxoviridae* human and avian models versus time of H1N1 Influenza A sequences. The colored lines are the reconstructed paths of the inferred phylogenetic tree that connect the root to each leaf (observed sequence), and the score versus inferred time is plotted also for the internal node (inferred) sequences. b) Log-likelihood differences of the MENB *Coronaviridae* model and a MENB model trained on the original Wuhan SARS-CoV-2 sequence versus time from 2019 December 26. The black line is a linear fit on the first 1,089 d (slope: $9\cdot 10^{-5}$, *P*-value: $10^{-9}$), the orange line is a linear fit of the last 180 d (slope: $3.5\cdot 10^{-4}$, *P*-value: $10^{-7}$). To ease the visualization of the increasing trend of the score difference in the last 180 d, daily averages of the score differences are plotted as orange points.

It is commonly accepted that the pandemics originated with a jump from avian to human hosts (Taubenberger et al. 2005). To compare the two hosts, we will use in our analysis the human and the avian model trained, for each host, on all the segments of influenza viruses excluding PB2 to avoid potential overfitting. Before assigning sequences to their host, we built a phylogenetic tree, on a random subsample of up to 20 sequences per year, using Nextstrain (Hadfield et al. 2018). Figure 9a shows the PB2 sequences log-probability difference between the influenza-human and influenza-avian MENB models at fixed viral family as a function of time since 1918. The log-probability difference allows classification of the host, similarly to the host classification task with MENB-H,V in Fig. 2 from the sequences sampled over time but also from the reconstructed roots along the phylogenetic tree. On left side of Fig. 9a, we observe that the 1918 PB2 segment (left side of Fig. 9a) has a larger score for avian model than for human ones, compatibly with the decoding of the avian host in supplementary fig. S13, Supplementary Material online.

The classification changes with time: as the virus evolve in contact with the human host, the model assigns to it higher log-probability differences, giving equal scores to human and avian origin around 1950. For more recent samples the model is more and more confident about the human classification. Quite remarkably, the log-probability score introduced here works as a sort of “molecular clock,” by steadily increasing as the virus adapts to the new host. Similar results are obtained also by a simple model only reflecting the nucleotide usage or also including the CpG forces (Greenbaum et al. 2014; supplementary fig. S17, Supplementary Material online), although in these cases the difference of log-probability between the two models is less pronounced and less dispersed across different sequences, confirming that taking into account 3-nucleotides (the simpler type of CpG context-dependence; Greenbaum et al. 2008; Takata et al. 2017), leads to a finer description of host adaptation.

As a final application of our MENB models, we turned to the SARS-CoV-2 virus. We wanted to check if we can see hints of host adaptation as for the 1918 H1N1 virus. This case is different from H1N1 as the original host of SARS-CoV-2 is currently unknown and subject of scientific debate (Andersen et al. 2020); we have therefore assumed that the original Wuhan sequence is representative of the (unknown) previous host and build its MENB model from this unique sequence, while building the MENB model for *Coronaviridae* in human host from the sequences used for the classification task in Section “MENB: A Model for Host and Viral Family Classification”. We then test the log-probability difference between the two models on the SARS-CoV-2 in human host from the sequences collected during the recent pandemic waves in Nextstrain (Hadfield et al. 2018).

The log-probability difference between the two models is plotted in Fig. 9b as a function of time for the first 1,100 d from the start of the 2020 pandemic. It shows a slow but steady adaptation to human nucleotide usage (black line, whose slope is significantly different from 0 with a *P*-value of $10^{-9}$). Quite surprisingly, the slope of the fitting line is larger for sequences collected in the last 6 months (data downloaded on 2023 June 30), suggesting an increase of the adaptation speed in the Omicron 23A variant that appeared in early January 2023 and rapidly took over the entire SARS-CoV-2 global population (Tan et al. 2022). It is worth noticing that the time over which the adaptation to the human host has been sampled is much smaller than that of the H1N1 strain, on such a short time scale adaption driven by nonsynonymous mutations with clear fitness advantages could result in a confounding signal.

### MENB Model Parameters Reflect Biologically Relevant Features

The MENB model is defined from a relatively low number of learnable parameters directly related to the usages of the motifs. It is therefore tempting to interpret these parameters, which can potentially give insight into the biology role of motifs.

**Fig. 10.**
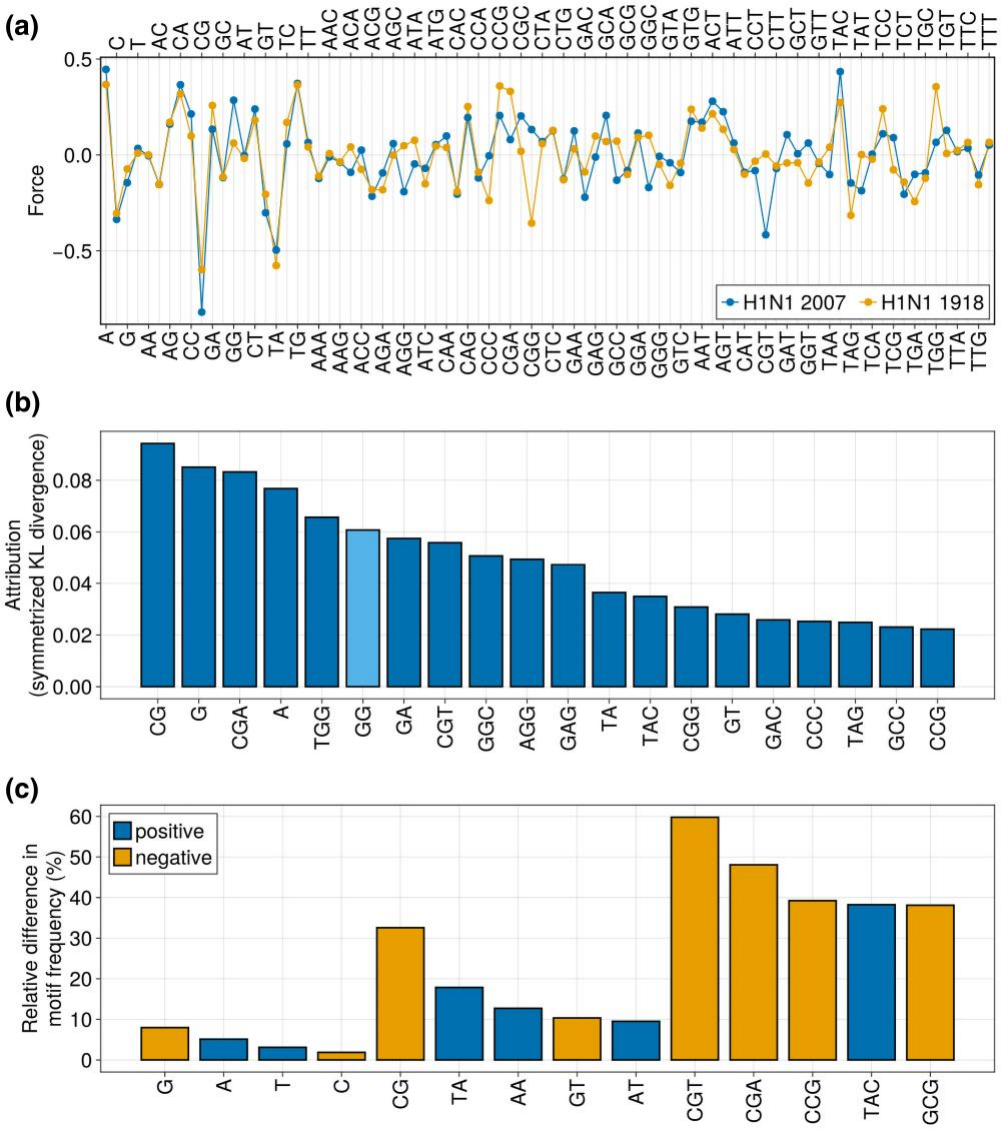
The learned parameters of MENB models can be directly visualized and interpreted. a) Plot of each of the 84 parameters (forces) learned by MENB models trained on all segments but PB2 of H1N1 Influenza A strains collected in 2007 (blue) and of the 1918 strain (orange). b) Attributions computed with the method of integrated gradients (Methods Section “Computation of the Partition Function and Related Quantities”) for the symmetrized KL divergence between the MENB models used in panel (a). To allow for an easier visualization, only the 20 parameters with the highest contributions (in absolute value) to the symmetrized KL divergence are shown. Light blue bars denote negative attributions. c) Relative difference in expected motif frequencies between the MENB models used in panel a (Methods Section “Computation of the Partition Function and Related Quantities”). Only the 5 top differences (in absolute value) are plotted for 2-mers and 3-mers. Light blue (orange) bars correspond to positive (negative) differences.

To showcase this, we considered two models trained on the PB2 segments of *Orthomyxoviridae* viruses: one (“H1N1 1918”) was trained on the sequence collected in 1918, the other (“H1N1 2007”) on 26 sequences collected in 2007. In Fig. 10a, we show the entire parameter profile of the two models. Nonzero parameters reflect the presence of selective forces that push up or down the number of the corresponding motif with respect to sequences generated uniformly at random. Considering the fact that the 1918 strain was likely of avian origin, the first interesting remark is an overall similarly of the force profile in the two cases, especially for nucleotides and dimers, which indicate that many of the force parameters did not significantly change during the adaptation to the human host.

The two dinucleotides with the largest negative forces are CpG, reflecting the well-known avoidance of CpG motifs, followed by UpA, another known avoided motif that is supposed to have a role in codon efficiency (Greenbaum et al. 2008; Lobo et al. 2009; Atkinson et al. 2014; Greenbaum et al. 2014). Moreover, the force in UpG and CpA motif is large and positive, likely due to the C→U and G→A mutational processes on, respectively, CpG and UpA motifs. These patterns are found on all the viruses and species analyzed here.

This observation points out an important concept in *k*-mer analyses of genetic sequences: the lack of one or more motif is necessarily compensated by an increase in abundance of other motifs, and vice-versa. In our framework, this is deeply connected to the *gauge choices* that have to be taken due to conservation of probabilities at single, di-, and tri-nucleotide levels and are discussed in more details in Methods Section “Gauge Choices for MENB Model.”

The differences in the parameter profile in Fig. 10a disclose the selective pressures on the nucleotide biases, dimers, and trimers driving the evolution of the viral sequence in the adaptation to the new host. The most striking differences between the 1918 and the 2007 viruses are the further decreases in the CpG force, as well as CGU motifs decrease, from a value around zero in 1918 to a negative value in 2007. An opposite evolution is observed for the GpG force increasing from zero to a positive value and for the CGG force which relaxes from a negative value toward zero in the 2007 (see also supplementary fig. S22, Supplementary Material online). The decrease in CpG forces confirms previous findings and what obtained with a simpler model containing only the CpG force, moreover the different behavior for the tri-nucleotide mirrors the context dependence of the CpG loss (Greenbaum et al. 2008, 2014).

A more rigorous way to study the evolution of the forces is to find the key parameters to discriminate the models inferred from the 1918 and 2007 sequences. This problem can be addressed within the framework of integrated gradients (Sundararajan et al. 2017): we compute the symmetrized KL divergence between the two MENB models as the sum of attributions, i.e. integrated gradients with respect to each parameter (more details about the procedure are given in Methods Section “Computation of the Partition Function and Related Quantities”; see supplementary fig. S20, Supplementary Material online for the comparison of symmetrized versus nonsymmetrized KL divergences). In Fig. 10b, we show the values for the top-20 attributions to the symmetrized KL divergence. Consistently with the force differences, we find that the largest attribution is on CpG dinucleotide, and several 3-nucleotides motifs containing CpG (CGA, CGU, CGG, and CCG) are present. The GpG and GpA and UpA dinucleotides and several related trinucleotides (TGG, GGC, GGC, CGG, GAG, TAC, and TAG) have large attributions too.

Once the inference of parameters is performed we compute the expected number of 1-, 2-, and 3-nucleotide motifs in a viral sequence according to the MENB models (Methods Section “Computation of the Partition Function and Related Quantities”), which (as shown in supplementary fig. S18, Supplementary Material online) should reproduce, by model construction, the motif frequencies in the data (Fig. 7a) (Wong et al. 2010). It is interesting to compare the force attributions in Flu evolution to the relative differences in motif frequencies (Fig. 10c). The relation between forces and frequencies is not direct as nucleotide or dinucleotide usage can, for instance, be driven also by tri-nucleotide forces. In agreement with the force attributions, CpG shows, among all dinucleotides in human-adapted H1N1 strains, the largest relative decrease in 2007 with respect to 1918 (Fig. 10c). Moreover, we observe more UpA and ApA dinucleotides with respect to the 1918 strain. As for 3-mers, the signal is dominated by decrease in usage of specific CpG-containing motifs; some other motifs such as TAC increase in frequency (Fig. 10c). It is important to notice that relative changes of 3-mers cannot be immediately compared with those of 2-mers, due to the fact that there are 64 different 3-mers and 16 2-mers and so individual 3-mers are in general rarer than individual 2-mers and largest changes are to be expected.

We next consider the main dataset used for virus and host classification (MENB-H,V models) to bring out similarities and differences in motif usage through the force parameters. The overall similarity of force profiles is again apparent, see supplementary fig. S14, Supplementary Material online, reflecting a direct cross contamination and adaptation through zoonotic transmissions or the presence of similar molecular mechanisms driving the adaptation of the viral sequences to the host. It is worth noticing that CpG force is the most negative force for all viral families and host, with different amplitudes. Moreover the difference between the human and avian CpG force value in the *Orthomyxoviridae* family is less pronounced than between the 1918 and the 2007 sequences shown in Fig. 10. This may be due to the fact that in supplementary fig. S14, Supplementary Material online the force represents a global value obtained from different sequences and strains (Greenbaum et al. 2008). Moreover, as shown in supplementary fig. S15, Supplementary Material online the force differences when including also 3-nucleotides, drive the model frequency of CpG motifs to smaller values in humans than in avian hosts as expected (Greenbaum et al. 2008; Lobo et al. 2009; Greenbaum et al. 2014).

In agreement with Fig. 3, larger differences are found across viruses than across hosts. The attributions and differences in motif usage depend quite strongly on both viral family and pair of host analyzed, as shown in supplementary figs. S15 and S16, Supplementary Material online, underlying the peculiarities of each viral family and host. This result further emphasizes the importance of inferring MENB models for each viral family and each host.

## Discussion

We have demonstrated, in a setting limited to four ssRNA viral families and three hosts, that our maximum-entropy approach can successfully be used to predict the viral family and the host of a virus from its sequence. Consistently with some recent empirical observations (Mock et al. 2020), we have shown viral sequences adapt to the host nucleotide usage under specific viral-family depending constraints. For the host-classification task, our interpretable MENB procedure exhibits competitive performance with VIDHOP, despite being much simpler and requiring much less defining parameters and, hence, training data. As expected from standard bias-variance tradeoff considerations (Posani et al. 2023), our method is less subject to the specific details of the training data and shows good out-of-class generalization properties, which have been benchmarked in several settings of increasing dissimilarities from the training data: (i) testing unknown sequences of the same family and host, (ii) training and testing partial segments of the viral genomes, (iii) testing sequences of known viral families but from new host taxa, (iv) testing sequences of known hosts but new ssRNA viral families or clades.

The above out-of-sample scenarios can be of direct applicability in practical cases, e.g. when only a portion of the genome is collected, or sequences of new viruses from unknown hosts are collected from environmental samples. A deeper benchmarking with a more complete train dataset should be performed to confirm and improve accuracy in decoding the host and to assess the ability of MENB-H,V to identify the closest viral families.

We have further shown that MENB models can be used for pandemic viral strains to decode the previous host in a viral host jump and to detect inter-host recombination/reassortment (Greenbaum et al. 2012; Dhama et al. 2020; Wille and Holmes 2020; Brierley and Fowler 2021; Di Gioacchino et al. 2021; MacLean et al. 2021). Our genome segmentation analysis shows large heterogeneity in the host classification accuracy of MENB models along the *Coronaviridae* and *Orthomyxoviridae* genomes, compatible both with that intra-host recombinations and reassortments and region-dependent selection pressures possibly exerted by the innate immune system and neutralizing antibodies. A detailed analysis of specific genomic regions in *Coronaviridae* and human strains shows that ORFS carries the best human fingerprint, while ORFN is rarely decoded as human. Similarly, in the influenza pandemic subfamilies, HA and NA have the best human fingerprints, while NP is not classified as human. The host decoding power of the MENB models is notably increased for *Coronaviridae* when training and testing (in the overlapping train-test setting) focus on the same genomic portion. Moreover a larger increase is obtained both in overlapping and nonoverlapping settings when testing on regions that adapt faster to the host, such as ORFS. In analogy to the 16S and 23S rRNA phylogenetic profiling in bacteria (Curry et al. 2022), such regions should be considered for a best host fingerprint. The efficiency of machine-learning models based on nucleotide motifs to predict the previous hosts of *Coronaviridae* of different strains from ORFS region was previously proven in Brierley and Fowler (2021). When these regions are not known a priori, a procedure consisting in inferring the host based on the information provided by all the regions (MaxSum) generally shows the best accuracy.

Finally, our framework can help design new sequences with given constraints on the nucleotide motif statistics and for the prediction of the viral genome evolution in a new host (Wille and Holmes 2020). As we have shown for H1N1 Influenza for which we have 100 years of sampled sequences at our disposal, the difference of the log-probabilities of the viral sequences computed in the new host model and the ones computed with the previous host model increases over time. These differences can be used as proxies for the degree of adaption of the viral sequences to their new host environment. Furthermore, the model’s parameters correspond to interpretable features in machine learning. The inferred profiles of 1-2-3 nucleotide forces, give useful sequences representations, directly interpretable in terms of PAMP-associated phenotypes (Tanne et al. 2015). By tracking their changes, one can shed light on molecular mechanisms at play in viral adaptation to human hosts. This is the case for the ZAP protein, which was shown to restrict CpG-rich sequences, after the discovery of CpG loss in adaptation to human hosts (Gao et al. 2002; Shackelton et al. 2006; Greenbaum et al. 2008, 2014; Di Giallonardo et al. 2017; Takata et al. 2017; Ficarelli et al. 2020). Here we have extended this profiling in a systematic way for all 1-2-3 nucleotide motifs, and extracted the parameters having largest differences (using the framework of the integrated gradients) in H1N1 evolution and among viral families and hosts. As an extension, it would be interesting to systematically compare the force parameters and their evolution in coding and noncoding part of the sequences. In a previous work, we considered a simple MENB model including only the CpG force and transition/transversion rates to score past synonymous mutations in SARS-CoV-2 from the original Wuhan sequence, and to predict future ones (Di Gioacchino et al. 2021). It would be interesting to repeat this study using the full MENB model presented here.

Our work could have several potential applications. The need to classify new viral sequences is likely to becomes more and more pressing in the near future, as new viral sequences continue to be discovered (Tisza et al. 2020; Edgar et al. 2022). While we have here benchmarked the MENB models in a limited setting of four ssRNA viral families and three hosts, extension to more families and hosts will be crucial in this perspective. The present study could in particular be extended to other viruses, notably DNA viruses and retroviruses, which are subjected to different selection pressures by the host. Moreover, the study could be extended to other host taxa including plants and insect, for which our method could characterize different features in motif usages. The fast and flexible host-detection algorithm introduced here can easily be integrated within metagenomics studies to infer the host of viruses, even if their sequences are quite different from the sequences used to train the algorithm.

Secondly, MENB models could help decipher the noncoding part of the human genome, also called “dark” matter. Recently, studies have pointed out viral origin and viral mimicry of some of repeats; for an early application of simplified MENB models to identify similarities between genomic regions and viral families by some of us, see Šulc et al. (2023).

MENB models can, moreover, be broadly used to study emerging pathogens and their adaptation to new hosts, as a support in surveillance studies (Wille and Holmes 2020). Also in these cases, the model can be optimized by adapting the dataset to train the model, e.g. by extending the ranges of hosts in the train data, or learning the model only from the segments representing the best fingerprints of the host. Modeling at the nucleotide level is necessary to capture some features of viral evolution that should be further combined with the inference of epistatic fitness landscapes of viral genomes combining synonymous and nonsynonymous mutations (Neher and Shraiman 2011; Minh et al. 2020; MacLean et al. 2021; Zeng et al. 2021). Finally, thanks to their generative properties underlined here, MENB models could be employed in RNA vaccine design for optimizing efficiency and minimizing rejection due to immunogenicity (Pardi et al. 2018; Zhang et al. 2023). By predicting how viruses adapt to their new host, we can better understand mechanisms that drive their adaptation and design intervention.

Future extensions of our work include more exhaustive benchmarking and possibly integration with methods using phylogeny (Minh et al. 2020) and gradient boosting machines (Babayan et al. 2018), or random forests (Brierley and Fowler 2021).

## Materials and Methods

### The Maximum Entropy Nucleotide Bias Model

In this section, we will first give a maximum-entropy derivation of the MENB model as given in the Equation in the Section “Results”. This will clarify why some of the parameters can be arbitrarily fixed as they are redundant (gauge choice) and we will discuss the specific choices made here. Finally, we will describe how by using statistical-physics methods all the computations involving the MENB model can be performed exactly and efficiently.

#### Maximum Entropy Justification

Consider a set of sequences observed (data), we want to find a probability distribution p(s) on the sequence space (model) such that: (i) the observed frequencies of nucleotides, 2-mers and 3-mers in the data match those expected by sampling sequences according to the model, and (ii) the entropy $-\sum_{s}p(s)\log\;p(s)$ is maximized. Therefore, the MENB model probability distribution maximizes the following quantity:


(1) $$\begin{array}{rl} & -\sum_{s}p(s)\log\;p(s)+\sum_{a\in S}f_{a}^{\left(1\right)}\left(\langle n_{a}(s)\rangle -n_{a}^{obs}\right)\\ & \;+\sum_{ab\in S}f_{ab}^{\left(2\right)}\left(\langle n_{ab}(s)\rangle -n_{ab}^{obs}\right)+\sum_{abc\in S}f_{abc}^{\left(3\right)}\left(\langle n_{abc}(s)\rangle -n_{abc}^{obs}\right) \end{array}$$

p(s) and the $f_{a}^{\left(1\right)},f_{ab}^{\left(2\right)}$, and $f_{abc}^{\left(3\right)}$. Here $\left\langle g(s)\rangle =\sum_{s}p(s)g(s\right)$, and quantities with the *obs* superscript are averages computed on the data sequences. By taking the functional derivative with respect to p(s), we obtain the functional form given in the Equation in the Section “Results”, where the Lagrange multipliers, that we also call force parameters, need to be fixed so that the observed frequencies of nucleotides, 2-mers and 3-mers in the data match those expected by sampling sequences according to the model. Following Greenbaum et al. (2014), this parameter inference can be performed by computing the partition function


(2) $$Z=\sum_{s\in S^{L}}e^{-E(s)}$$

(3) $$E(s)=-\left(\sum_{a\in S}f_{a}^{\left(1\right)}n_{a}(s)+\sum_{ab\in S}f_{ab}^{\left(2\right)}n_{ab}(s)+\sum_{abc\in S}f_{abc}^{\left(3\right)}n_{abc}(s)\right)$$

that normalizes the probability distribution in the Equation in the Section “Results” and using it to estimate the quantities $\left\langle n_{a}(s)\right\rangle$, $\left\langle n_{ab}(s)\right\rangle$, $\left\langle n_{abc}(s)\right\rangle$. Finally, a root-finding algorithm such as the Newton–Raphson method can be used to find the correct values for the parameters. Optionally, the observed quantities $n_{a}^{obs},n_{ab}^{obs}$, and $n_{abc}^{obs}$ can be regularized by adding pseudocounts to avoid parameter divergences for small counts.

#### Gauge Choices for MENB Model

The MENB model specifies a probability distribution over sequences of length *L*. As such, any change of parameters that does not change the probability of any sequence does not have any effect and it is called a gauge degree of freedom. For instance, we can send $f_{a}^{\left(1\right)}\to f_{a}^{\left(1\right)}+K$ and, for any value of *K*, this change does not impact the probability of any sequence as it can be readily shown using the fact that $\sum_{a\in S}n_{a}(s)=L$. As a consequence, we are free to choose a value for *K* so that, for instance, $f_{T}^{\left(1\right)}=0$, or so that $\sum_{a\in S}f_{a}^{\left(1\right)}=0$.

The presence of gauge degrees of freedom stems from the fact that there are many ways of choosing the 84 force parameters in Equation in the Section “Results” so that the observed frequencies of nucleotides, 2-mers and 3-mers in the data match those expected from to the model. Indeed, although this requirement can be written as a set of 84 equations, some of them are not independent because of the following considerations:


(4) $$\begin{array}{rl} & \sum_{a\in S}n_{a}(s)=L\\ & \sum_{ab\in S}n_{ab}(s)≃L\\ & \sum_{a\in S}n_{ax}(s)≃n_{x},\;\sum_{a\in S}n_{xa}(s)≃n_{x}\;\forall \;x\in S\\ & \sum_{abc\in S}n_{abc}(s)≃L\\ & \sum_{ab\in S}n_{abx}(s)≃n_{x},\;\;\sum_{ab\in S}n_{axb}(s)≃n_{x}\sum_{ab\in S}n_{xab}(s)≃n_{x}\;\;\forall \;x\in S\\ & \sum_{a\in S}n_{xya}(s)≃n_{xy},\;\sum_{a\in S}n_{axy}(s)≃n_{xy}\;\forall \;x,y\in S \end{array}$$

Where the symbol ≃ means that the condition is respected in the large-*L* limit, which is the relevant case for all sequences considered in this work. This set of equations can be used to fix the gauge degrees of freedom (“choose the gauge”), and we do so in this work by choosing a gauge where the maximum number of parameters is set to zero, that we call parsimonious gauge, or by choosing a gauge where there is no arbitrary symmetry breaking among the model parameters, that we call zero-sum gauge.

For the parsimonious gauge, we decide to set to zero all forces of the form $f_{T}^{\left(1\right)}$, $f_{Tx}^{\left(2\right)}\;\forall \;x\in S$, $f_{xT}^{\left(2\right)}\;\forall \;x\in S$, $f_{TTT}^{\left(3\right)}$, $f_{TTx}^{\left(3\right)}\;\forall \;x\in S$, $f_{TxT}^{\left(3\right)}\;\forall \;x\in S$, $f_{xTT}^{\left(3\right)}\;\forall \;x\in S$, $f_{Txy}^{\left(3\right)}\;\forall \;x,y\in S$, $f_{xyT}^{\left(3\right)}\;\forall \;x,y\in S$. Therefore, nonzero *T*-containing forces only have the form $f_{xTy}$ with x,y∈S. This means that the effective number of free parameters to be inferred goes from 84 to 48.

The parsimonious gauge is particularly useful to speed up the inference process. For the sake of interpretation, after inference, it is preferable to turn to the zero-sum gauge, defined by the following set of equations:


(5) $$\begin{array}{rl} & \sum_{a\in S}f_{a}^{\left(1\right)}=0\\ & \sum_{a\in S}f_{xa}^{\left(2\right)}=\sum_{a\in S}f_{ax}^{\left(2\right)}=0\;\forall \;x\in S\\ & \sum_{a\in S}f_{xya}^{\left(3\right)}=\sum_{a\in S}f_{axy}^{\left(3\right)}=0\;\forall \;x,y\in S. \end{array}$$

#### Computation of the Partition Function and Related Quantities

A remarkable characteristic of the MENB model is that the partition function *Z* given in Equation (2) can be computed exactly in a time that scales linearly with the length of the sequence *L* using the so-called transfer matrix method, well known in statistical physics. This method has been already described for a similar problem in Greenbaum et al. (2014) (Supplementary material), and the only difference in this case is that the matrices also contain a term that accounts for the 3-body interaction.

Once the partition function of a MENB model is computed, we have immediate access to a wealth of relevant quantities. In particular, we can compute the expected number of ℓ-mers *M* as


(6) $$\langle n_{M}(s)\rangle =\frac{\partial }{\partial f_{M}^{\left(\ell \right)}}\log\;Z,$$

which is the main quantity used to produce Fig. 10c.

Another relevant quantity is the KL divergence between two models, $p_{1}$ and $p_{2}$. It can be written as


(7) $$D_{\mathrm{KL}}(p_{1},p_{2})=\sum_{s}p_{1}(s)\log\;\left(\frac{\;p_{1}(s)}{p_{2}(s)}\right)=\log\;Z_{2}-\log\;Z_{1}+\sum_{s}p_{1}(s)(E_{2}(s)-E_{1}(s)).$$

where $\log\;Z_{1}$ and $\log\;Z_{2}$ can be computed exactly with the transfer matrix method, and to compute the last term on the r.h.s. of Equation (7) we define


(8) $$Z_{12}(\lambda )=\sum_{s}e^{-E_{1}(s)+\lambda (E_{2}(s)-E_{1}(s))},$$

and we have


(9) $$\sum_{s}p_{1}(s)(E_{2}(s)-E_{1}(s))={\frac{\partial }{\partial \lambda }\log\;Z_{12}(\lambda )|}_{\lambda =0}.$$

From the KL divergence, we can compute the attributions shown in Fig. 10b. Following Sundararajan et al. (2017), we consider two MENB models defined by the force parameters $f_{1}$  $f_{2}$. We will use the notation $D_{\mathrm{KL}}(f_{1},f_{2})$ to denote the KL divergence between the models with parameter $f_{1}$ and $f_{2}$. Thanks to the fundamental theorem of calculus for line integrals, and using $D_{\mathrm{KL}}(f_{1},f_{1})=0$, we get


(10) $$\begin{array}{r} D_{\mathrm{KL}}(f_{1},f_{2})=\sum_{i}(f_{1,i}-f_{2,i})\int_{0}^{1}\nabla_{i}D_{\mathrm{KL}}(f_{2}+t(f_{1}-f_{2}),f_{2})\;dt. \end{array}$$

The individual terms of the sum in this equations are the attribution plotted, after rescaling for the total KL divergence, in Fig. 10b and supplementary fig. S16, Supplementary Material online. As a final remark, we notice that the attributions depends on the gauge used. In this work, we always computed attributions in the zero-sum gauge, and we observe that if the parameters $f_{1}$ and $f_{2}$ are from models in the zero-sum gauge, then Equation (5) still hold for $f_{1}+t(f_{1}-f_{2})$, and so the path of models used in Equation (10) preserve the zero-sum gauge.

#### MaxSum Inference Method

A procedure called MaxSum is used to investigate the possibility of improving accuracy performance of full-length genomes. For each viral sequence s, we partition the genome into $2^{d}$ continuous segments $s_{i}$, where d=1,2,3, or 4 is the depth of the partition. For the sake of simplicity, we focus on MENB-H only, a similar approach can be used for MENB-H|V and MENB-H,V.

We combine the probabilities that the different segments $s_{i}$ have been generated from the host-dependent model p(s|h) into a single quantity


$$Q(s_{1},s_{2},\ldots |h,\beta )=\sum_{i}[\;p(s_{i}|h){]}^{\beta },$$

where *β* is a real number we choose at our convenience (see below). Next, we maximize *Q* over the host *h* to make our prediction.

The choice of *β* allows us to decode the host over different scenarios. The limiting cases are (i) β→∞: *h* is the host that is most likely to be associated with the segment with the highest probability, regardless of its ability to explain the statistics of the other segments, and (ii) β→−∞: *h* is the host providing the highest score to the worst segment (with the lowest probability among all segments), regardless of the scores of the other better segments. Informally speaking, the latter choice aims at finding a host roughly compatible with all segments and may be appropriate in the absence of reassortment for instance, while the former demands a very good match with (at least) one segment and can be useful to unveil complex evolutionary scenarios.

As these two extreme cases can be subject to statistical fluctuations, we report results obtained for β=1 in the main text, and results obtained when β→0 ( which corresponds to maximizing over *h* the sum of the logarithms of the probabilities) in supplementary fig. S9, Supplementary Material online.

### Metropolis–Hasting Algorithm to Evolve Sequences

To evolve sequences at fixed amino-acid content, we use the Metropolis–Hasting Monte Carlo algorithm. We start from an input sequence and the model Equation in the Section “Results” defined by a set of force parameters. In Fig. 8, the model is defined by the forces inferred from the 1918 flu except the $f_{CpG}$ obtained from the human genome (Greenbaum et al. 2014). To evolve the sequence at each step of the algorithm, a synonymous mutation is proposed and the difference in energy ΔE=E(s(t+1))−E(s(t)) (Equation 3) is computed with respect to the previous sequence. The mutation is always accepted if ΔE<0 and accepted with probability $e^{-\Delta E}$ if ΔE>0.

## Supplementary Material

msaf127_Supplementary_Data

## Data Availability

All sequence data have been collected from the BV-BRC database (Olson et al. 2022). After discarding short viral sequences (length lower than 1,000 bases), we selected the pairs of host and viral family so that each viral family has at least 100 sequences annotated with each host chosen. To train MENB models, 1,200 sequences were selected from the Zenodo file, 100 for each viral–host under study. 12 MENB-H|V, (or equivalently MENB-H,V) were trained for each viral–host combination. For MENB-H, three models for each host were trained, considering 400 sequences independently of the four viral families. An independent set of 600 genomes, 50 for each viral–host pair, has been chosen to test the model, which for MENB-H|V are taken separately for each viral family (150 sequences at the time). We have repeated the procedure three times to average the results and to provide an estimate of the standard deviations. The main dataset to train and test the MENB models is described in supplementary fig. S1, Supplementary Material online and is available at https://zenodo.org/doi/10.5281/zenodo.10050076. It is important to notice that we discarded Influenza A sequences collected after 2009 as if we consider them the database become dominated by strains of the H1N1 “swine flu” not yet adapted to the human host (Garten et al. 2009) (see Fig. 6). The SARS-CoV-2 data taken during the Coronavirus pandemic are heavily biased, both geographically (a large fraction of the sequences are collected in a small number of countries) and temporarily (the rate of sequence collection increased steadily in the first months of the pandemics). To address this issue, we used a curated dataset of sequences collected by Nextstrain (Hadfield et al. 2018) where sequences are subsampled to reduce biases from different geographic regions and time periods, and most of the sequences are collected in the last 6 months. To allow exact reproducibility of our results we the data we used (downloaded on 2023 June 30 from https://nextstrain.org), together with the sequence data at https://zenodo.org/doi/10.5281/zenodo.10050076. models is written in Julia and publicly available in the GitHub repository at https://github.com/adigioacchino/MaxEntNucleotideBiases.jl. A Snakemake pipeline to train and test the MENB models on the data used here is available at https://github.com/adigioacchino/MENB_snakemake. Additionally, for readers use, a notebook to test new sequences with the main dataset models, as well as an example on how to train, test, and sample for any generic virus and host dataset is available at https://github.com/IvanLec/Viral_Host_Adaptation-Testing_Training_and_Sampling. Additional datasets were composed to study the host fingerprints in *Coronaviridae* and *Orthomyxoviridae* genomes for specific strains and specific regions downloading sequences from PMC NCBI https://pmc.ncbi.nlm.nih.gov/. Two test sets of 20 sequences for virus infecting humans were considered for the analysis on full genomes and on ORF1ab, S, M, N and 3’ to 5’UTR for *Coronaviridae* (MERS, HCoVs, SARS-CoV-2) and HA, NA, PB2, and NP for *Orthomyxoviridae* Influenza A H1N1, H2N2, H3N2, and H5N2 (supplementary figs. S2a, S6b, S12, and S13, Supplementary Material online). A set for *Coronaviridae* composed by 30 SARS-CoV-2 for humans, 30 *Porcine Deltacoronavirus* and 30 *Infectious bronchitis virus* (considering only chickens for avian). (Fig. 6a, supplementary fig. S2b and tables S3 and S4, Supplementary Material online) A dataset for 1 sequence of H1N1 Influenza A Human, and 2 of H3N2 Influenza A Swine, was also used to compare MENB decoding with phylogenetic analysis Fig. 6b. The dataset of viral sequences sampled from new hosts (supplementary table S2D and fig. S10, Supplementary Material online) contain five sequences infecting camel, bovine, canine, rodent, feline, and bat species for *Coronaviridae*, bovine, feline, and canine for both *Picornaviridae* and *Orthomyxoviridae* and finally bovine, insect and sheep species for *Flaviviridae*. For *Orthomyxoviridae*, only the PB2 segments was used. The dataset of viral sequences sampled from new viral families (supplementary table S2C and fig. S11, Supplementary Material online) was composed by 30 sequences of *Caliciviridae* (10 for each human, avian and swine host, with only chicken as avian species) and a set of 90 sequences made by 30 sequences of *Paramyxoviridae* for avian (chicken), 30 of *Rhabdoviridae* for humans, and 30 of *Arteriviridae* for swine. These sequences were downloaded from PMC NCBI while the taxonomic tree information in supplementary fig. S11b, Supplementary Material online were obtained from NCBI Taxonomy https://www.ncbi.nlm.nih.gov/taxonomy. For all the additional datasets the viral and host probabilities are averaged over the MENB models built on the three sub-samples of train data in the main datasets. Genomes of the main dataset (supplementary fig. S1, Supplementary Material online) have been divided in halves, fourths, eights, and sixteenths to create new training and test datasets for partial genomes. We have considered six settings for train and test data composition, described in Fig. 11a. *Full Test:* partial genomes are used only in train data. *Full Train:* partial genomes are used only in test data. *Overlapping Train-Test:* the same portion is used in the test and train datasets. *Nonoverlapping Train*<*Test* and *Nonoverlapping Train*>*Test:* Partial nonoverlapping genomes are used in train and test data with the smallest portion in the train or test sets. Partial genomes. a) Illustration example, for second regions, of train and test genomic portions for a 4-fold genomic. The portions taken in the train set are underlined in blue and the ones in the test set in green. *Full Test*: train data from the second portion, over four, of the genomes, test data from the full genomes. *Full Train*: Train data from full genomes, test data from the second portions of them. *Non Overlapping Train*<*Test*: train data from the second portions of the genomes, test data from complementary regions. *Non Overlapping Train*>*Test*: test data from the second portions of the genomes, train data on the complementary portions. *Overlapping Train-Test*: the model is trained and tested on the same portions b) *Random segmentation* taking 50% genomes (randomly chosen) inside each train sequences, and testing on 25% randomly chosen segments of each test set sequence. Since sequences have different lengths (see in particular supplementary table S1, Supplementary Material online), and no alignment was performed, the concept of overlapping or nonoverlapping portions must be considered in a broad sense, as we do not verify that the same coding regions are included or excluded in the train and test datasets. Partitions in the train and test data of segments of different percentage of genomes length: 100%, 50%, 25%, 10%, 5%, 2.5%, or 1%, randomly located along the genomes both for train and test sets shown in Fig. 11b.

## Data Availability

As described in the Method Section the data underlying this article are available at https://zenodo.org/doi/10.5281/zenodo.10050076. Codes are available at the GitHub repositories at https://github.com/adigioacchino/MaxEntNucleotideBiases.jl and https://github.com/IvanLec/Viral_Host_Adaptation-Testing_Training_and_Sampling.
