# Supplementary material for: Deciphering the Code of Viral-Host Adaptation Through Maximum-Entropy Nucleotide Bias Models
Source: Mol Biol Evol. 2025 Jun 3;42(6):msaf127. doi: 10.1093/molbev/msaf127 (PMC12205363; doi:10.1093/molbev/msaf127)
Supplement: msaf127_Supplementary_Data [file msaf127_supplementary_data.pdf]

## Supplementary figures

# Deciphering the code of viral-host adaptation through maximum-entropy nucleotide bias models

Andrea Di Gioacchino, Ivan Lecce, Benjamin D. Greenbaum, Rémi Monasson, Simona Cocco

| **Train Average Length** | Human | Swine | Avian |
|---|---|---|---|
| Corona | 29770±1224 | 27500±1677 | 27646±82 |
| Flavi | 10254±56 | 11666±735 | 10797±306 |
| Picorna | 7290±173 | 7624±418 | 8260±847 |
| Orthomyxo | 13346±218 | 13312±257 | 13335±548 |
| All | 15165±8727 | 15025±7553 | 15010±7532 |
| **Test Avarage Length** | Human | Swine | Avian |
| Corona | 29676±1235 | 27316±2092 | 27652±69 |
| Flavi | 10284±530 | 11714±709 | 10774±345 |
| Picorna | 7314±183 | 7611±407 | 8223±831 |
| Orthomyxo | 13352±116 | 13260±507 | 13380±243 |
| All | 15156±8677 | 14976±7506 | 15007±7539 |

Table 1: **Viral length information for MENB datasets.** Average length of viral sequences, used to train and test MENB models (for datasets details see Suppl. Fig. 1).

| **Min Length** | Human | Swine | Avian |
|---|---|---|---|
| Corona | 346 | 271 | 394 |
| Flavi | 163 | 184 | 172 |
| Picorna | 211 | 130 | 261 |
| Orthomyxo | 347 | 302 | 319 |
| All | 194 | 170 | 207 |

| **Min Percentage** | Human | Swine | Avian |
|---|---|---|---|
| Corona | 1.1% | 0.9% | 1.4% |
| Flavi | 1.5% | 1.5% | 1.5% |
| Picorna | 2.8% | 1.6% | 2.6% |
| Orthomyxo | 2.5% | 2.2 % | 2.3% |
| All | 0.6 % | 0.6 % | 0.7% |

Table 2: **Minimum length in nucleotides and minimum length percentage values needed to train the models**. The minimum length of training sets has been computed by averaging the nucleotide motif frequencies for each category shown in the table and by taking the inverse of the minimum: $L_{min} \propto \frac{1}{min\{f_m\}}$. The percentage has been obtained from the quantity $\frac{L_{min}}{\langle L \rangle}$ where $\langle L \rangle$ represents the average length of sequences computed for each training set (shown in Suppl. Table 1).

**A**

*Corona*

| Human | Training | Test |
|---|---|---|
| MERS-CoV | 115 | 51 |
| SARS-CoV2 | 8 | 10 |
| HCoV-NL63 | 34 | 23 |
| HCoV-OC43 | 109 | 48 |
| HCoV-HKU1 | 11 | 8 |
| HCoV-229E | 17 | 7 |
| PDCoV | 3 | 1 |
| CoV-EMC-2C | 1 | 1 |
| BCoV-EN1 | 2 | 0 |
| HECoV-4408 | 0 | 1 |

| Avian | Training | Test |
|---|---|---|
| IBV | 279 | 137 |
| Avian CoV | 20 | 11 |
| Duck CoV | 1 | 2 |

| Swine | Training | Test |
|---|---|---|
| PEDV | 217 | 106 |
| PDCoV | 59 | 36 |
| PHEV | 7 | 3 |
| SeCoV | 2 | 0 |
| TGEV | 13 | 4 |
| PRCV | 1 | 0 |
| PToV | 1 | 1 |

*Flavi*

| Human | Training | Test |
|---|---|---|
| DENV-1 | 64 | 40 |
| DENV-2 | 65 | 24 |
| DENV-3 | 46 | 27 |
| DENV-4 | 18 | 10 |
| HCV | 71 | 32 |
| YFV | 5 | 3 |
| ZIKV | 19 | 9 |
| HPgV | 7 | 1 |
| WESSV | 1 | 0 |
| AHFV | 1 | 0 |
| WNV | 3 | 1 |
| TBEV | 0 | 2 |
| POWV | 0 | 1 |

| Avian | Training | Test |
|---|---|---|
| WNV | 40 | 19 |
| DEDSV | 9 | 9 |
| TMUV | 234 | 103 |
| DHV | 5 | 7 |
| ITV | 3 | 0 |
| Duck flavivirus TA | 2 | 1 |
| USUV | 1 | 2 |
| HPgV-1 | 2 | 1 |
| BYDV | 1 | 2 |
| Flavivirus muscovy | 1 | 2 |
| MDRV | 1 | 2 |
| HPgV-2 | 1 | 2 |

| Swine | Training | Test |
|---|---|---|
| CSF | 147 | 77 |
| JEV | 63 | 29 |
| BDV | 3 | 3 |
| APPV | 68 | 35 |
| PPgV | 13 | 4 |
| BVDV-2 | 2 | 1 |
| LindaV | 4 | 1 |

*Picorna*

| Human | Training | Test |
|---|---|---|
| EVs | 96 | 48 |
| Coxsackievirus | 72 | 40 |
| Human poliovirus | 18 | 10 |
| HRVs | 88 | 37 |
| ECHO | 14 | 6 |
| PeVs | 9 | 3 |
| AiV | 1 | 3 |
| Cosavirus | 2 | 1 |
| Cardiovirus | 0 | 0 |
| Saffold virus | 0 | 1 |

| Avian | Training | Test |
|---|---|---|
| Avihepatovirus | 16 | 12 |
| Avisivirus | 3 | 5 |
| Megrivirus | 40 | 20 |
| DVH | 153 | 74 |
| Anativirus | 13 | 4 |
| TVH | 4 | 2 |
| Pigeon mesivirus | 3 | 0 |
| Pigeon picornavirus | 2 | 0 |
| AEV | 3 | 2 |
| Orivirus | 6 | 0 |
| ChPV | 11 | 4 |
| Sicinivirus | 33 | 18 |
| Goose picornavirus | 7 | 4 |
| Melegrivirus | 3 | 0 |
| Gallivirus | 3 | 3 |
| Duck picornavirus | 0 | 2 |

| Swine | Training | Test |
|---|---|---|
| FMDV | 33 | 11 |
| EVs | 58 | 28 |
| PKoV | 55 | 33 |
| Porcine teschovirus | 13 | 3 |
| PSV | 36 | 28 |
| EMCV | 7 | 6 |
| SVV | 97 | 40 |
| Pasivirus | 1 | 1 |
| SVD | 0 | 11 |

*Orthomyxo*

| Human | Training | Test |
|---|---|---|
| 1990-1999 | 173 | 66 |
| 1980-1989 | 41 | 28 |
| 1970-1979 | 38 | 39 |
| 1960-1969 | 19 | 9 |
| 1950-1959 | 16 | 5 |
| 1940-1949 | 3 | 1 |
| 1930-1939 | 10 | 2 |

| Avian | Training | Test |
|---|---|---|
| 1990-1999 | 116 | 63 |
| 1980-1989 | 98 | 41 |
| 1970-1979 | 67 | 32 |
| 1960-1969 | 5 | 8 |
| 1950-1959 | 10 | 6 |
| 1940-1949 | 0 | 0 |
| 1930-1939 | 4 | 0 |

| Swine | Training | Test |
|---|---|---|
| 1990-1999 | 54 | 23 |
| 1980-1989 | 62 | 40 |
| 1970-1979 | 172 | 81 |
| 1960-1969 | 4 | 4 |
| 1950-1959 | 2 | 0 |
| 1940-1949 | 2 | 0 |
| 1930-1939 | 4 | 2 |

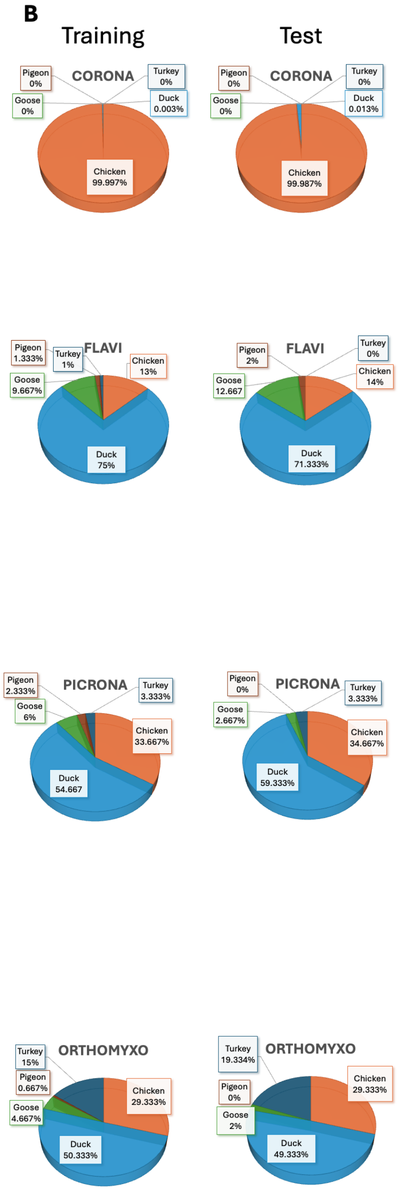


Figure 1: **Datasets used to train and test MENB models**. **A**:Composition of the train data sets (300 sequences) and test data-set (150 sequences), shown for each virus-host combination used to build the MENB-H,V and MENB-H|V models. The same sets, grouped for hosts form the datasets of the MENB-H model. Viral specifications are given for *Coronaviridae*, *Flaviviridae* and *Picornaviridae*, while for *Orthomyxoviridae*, being all Influenza A viruses, time labels in decades are shown. The most predominant virus for train or test in a given set has been highlighted in red. **B**: Statistics of host for avian species considered for all viral families.

**A**

| Human Coronaviridae | Test |
|---|---|
| MERS | 5 |
| HCoV-OC43 | 5 |
| HCoV-NL63 | 5 |
| Sars-CoV-2 | 5 |

| Human Orthoyxoviridae | Test |
|---|---|
| H1N1 | 5 |
| H2N2 | 5 |
| H3N2 | 5 |
| H5N1 | 5 |

**B**

| Host | Coronaviridae | Training-Test |
|---|---|---|
| Avian | IBV | 30 |
| Human | Sars-CoV2 | 30 |
| Swine | PDCoV | 30 |

**C**

| Human | Test |
|---|---|
| EKV | 3 |
| Mundri | 1 |
| Duvenhage | 3 |
| bat lyssavirus | 2 |
| Lyssavirus rabies | 15 |
| Chandipura virus | 3 |
| Jurona virus | 1 |
| Le Dantec virus | 1 |
| BASV | 1 |
| Norovirus | 10 |

| Avian | Test |
|---|---|
| NDV | 12 |
| APMV-1 | 15 |
| Avian Orthoavulavirus | 3 |
| Chicken calicivirus | 10 |

| Swine | Test |
|---|---|
| PRRS | 30 |
| Porcine sapovirus | 10 |

**D**

*Corona*

| Camel | Test |
|---|---|
| MERS | 5 |

| Bat | Test |
|---|---|
| BtCoV | 5 |

| Canine | Test |
|---|---|
| Canine respiratory coronavirus | 5 |

| Rodent | Test |
|---|---|
| RtAs-CoV | 1 |
| RtAp-CoV | 1 |
| RtNn-CoV | 1 |
| RCoV | 1 |
| RtMruf-CoV-2 | 1 |

| Bovine | Test |
|---|---|
| Bovine coronavirus | 5 |

| Feline | Test |
|---|---|
| Feline coronavirus | 5 |

*Picorna*

| Bovine | Test |
|---|---|
| Bovine picronavirus | 1 |
| BKoV | 3 |
| Aichivirus B | 1 |

| Feline | Test |
|---|---|
| Feline kobovirus | 5 |

| Canine | Test |
|---|---|
| Canine picodicistrovirus | 3 |
| Canine kobovirus | 2 |

*Orthomyxo*

| Bovine | Test |
|---|---|
| Infleunza D | 5 |

| Feline | Test |
|---|---|
| Influenza A H3N2 | 3 |
| Influenza A H5N6 | 2 |

| Canine | Test |
|---|---|
| Influenza A H9N2 | 1 |
| Influenza A H3N2 | 4 |

*Flavi*

| Bovine | Test |
|---|---|
| Bovine viral diarrhea | 5 |

| Sheep | Test |
|---|---|
| Louping ill virus | 5 |

| Insect | Test |
|---|---|
| Cell fusing agent virus | 5 |

Figure 2: **Additional datasets for *Coronaviridae*, *Orthomyxoviridae*, new hosts and new viral families**. **A**: Human *Coronaviridae* and *Orthomyxoviridae* test sets for host predictions in full genomes (used in Fig. 5 and in specific regions (used in Suppl. Figs. 12, 13). Among the sequences analyzed, 1 MERS, HCoV-OC43, 2 H1N1, 3 H2N2 and 5 H3N2 coincided with the Test set ones from Suppl. Fig. 1. **B**: Dataset of *Coronaviridae* used for specific region analysis: ORF1ab, S, M, N, or 3'-5' UTR using them as test set on the full genome model, or training a new model on this data set in non-overlapping (used in Suppl. Table 3) or full genome in the train data ( used in Suppl. Table 4) settings. When using these data to train a new model, for each host, 10 sequences were used to train the model and other 10 to test, averaging results over three sub-samples of train/test partitions. **C**: New viral sequences of *Paramyxoviridae* in avian, *Rhabdoviridae* in humans and *Arteriviridae* in swines. Sequences of *Caliciviridae* for all the three hosts are separated by blue lines, used in Suppl. Fig. 11. **D**: Dataset for new hosts infected by viruses of the four main viral families, used in Suppl. Fig. 10.

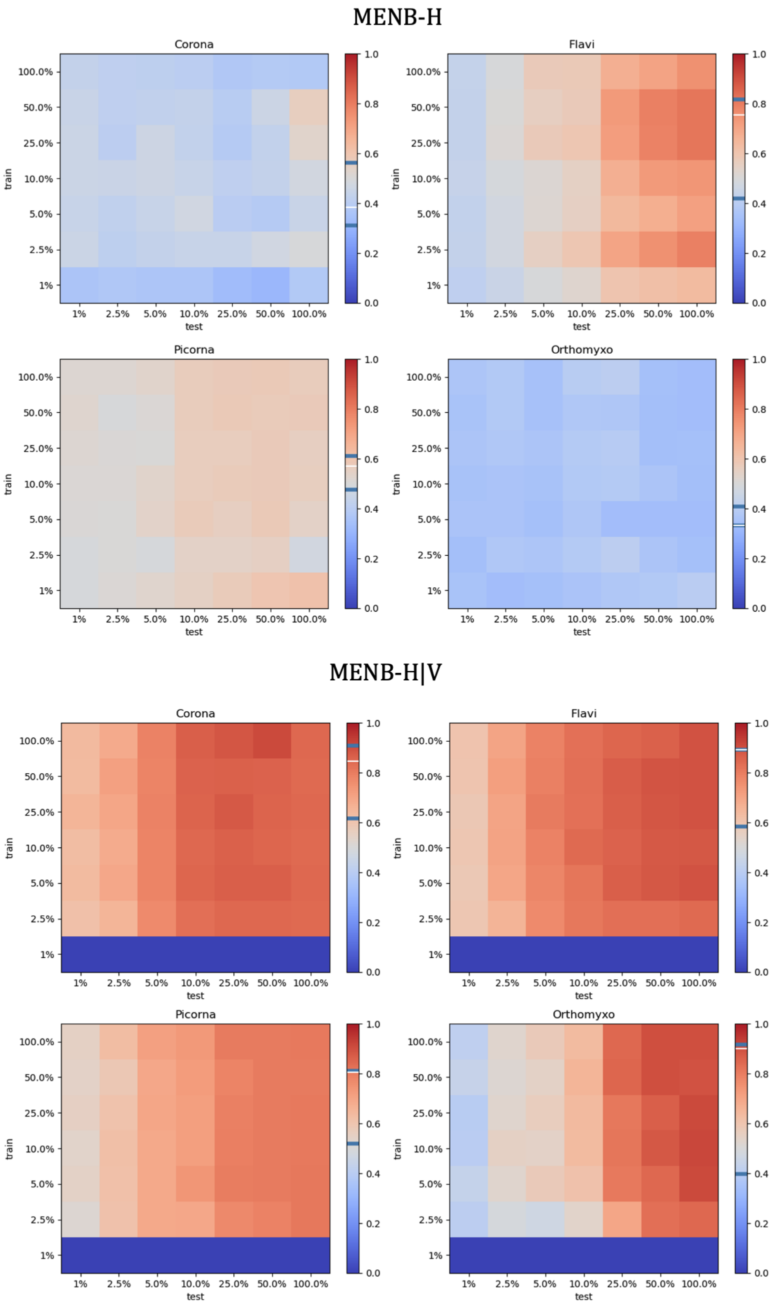


Figure 3: **MENB-H and MENB-H|V accuracy for host prediction from random genomic segments in train or test sets.** For both train and test data, genome portions ranging from 1% to 100% (full genome) have been taken. The dark blue lines at 1% of train for MENB-H|V indicate were the model cannot be trained, consistent with the minimal length shown in Suppl. Table 2. In the color bars, a white line shows the full-model prediction (top corner 100% Train-Test) while the two in light blue give the minimal and maximal accuracies.

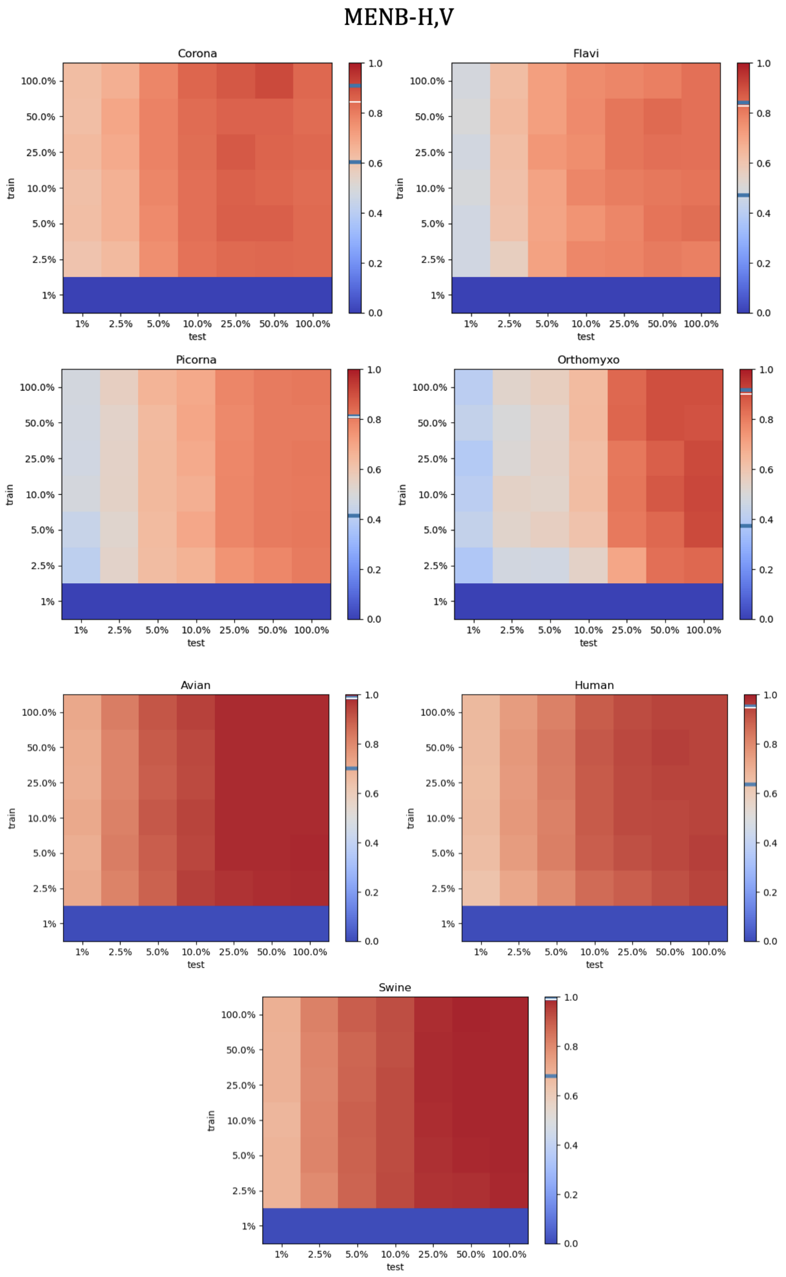


Figure 4: **MENB-H,V for host and viral family prediction from random genomic segments in train or test sets.** Top: Host predictions. Bottom: Viral family prediction. The dark blue for 1% indicates that the model cannot be trained

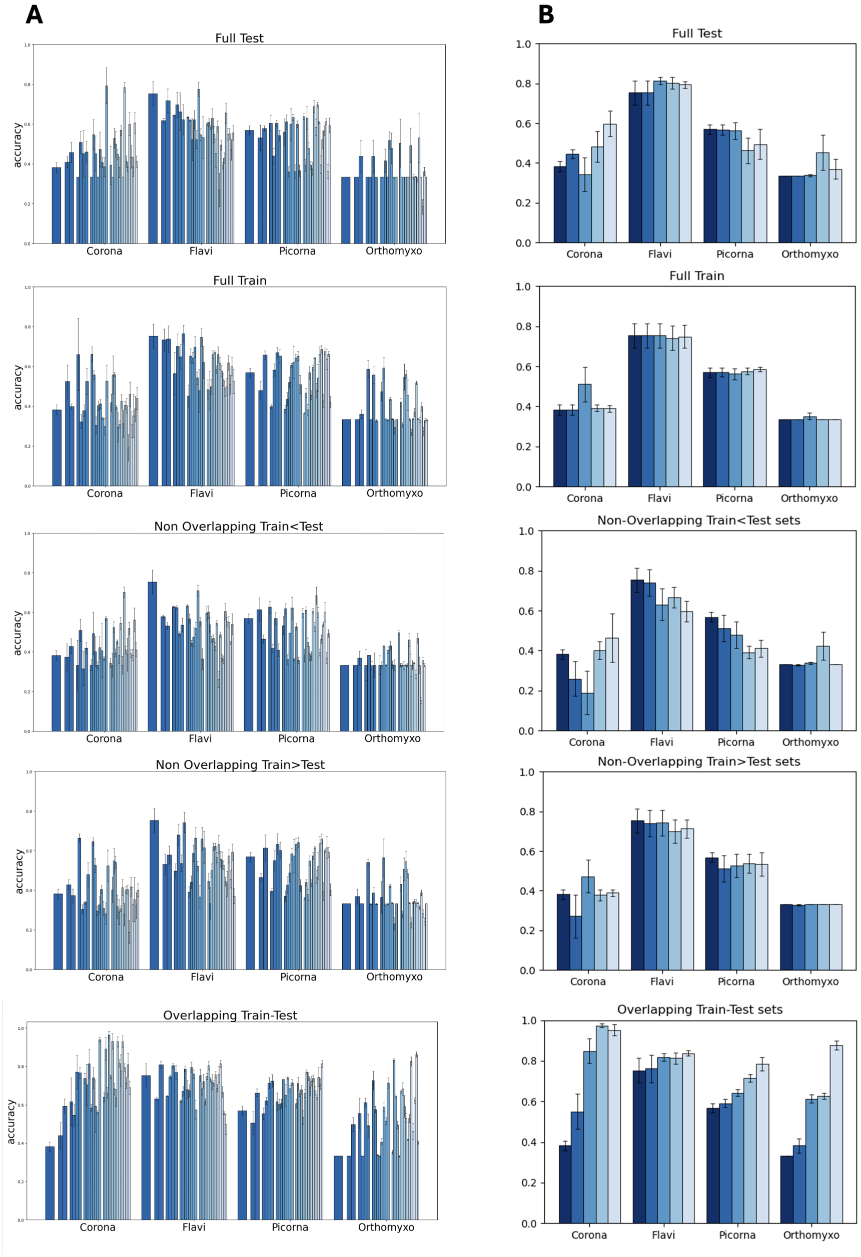


Figure 5: **MENB-H accuracies host prediction from genomic segments in different train-test settings**. **A**: Accuracy over all the segments of genomes at each binary division of sequences until a depth of four (16 segments) in different analysis procedure: Full genomes in the test set, Full genomes in the train set, Non-Overlapping segments with segments in Train $<$ then the ones in the test, Non-Overlapping segments with segments in Train$>$ than the ones in Test, Overlapping Train-Test segments (see Methods 5.3). **B** MaxSum at $\beta = 1$ for the same cases.

.

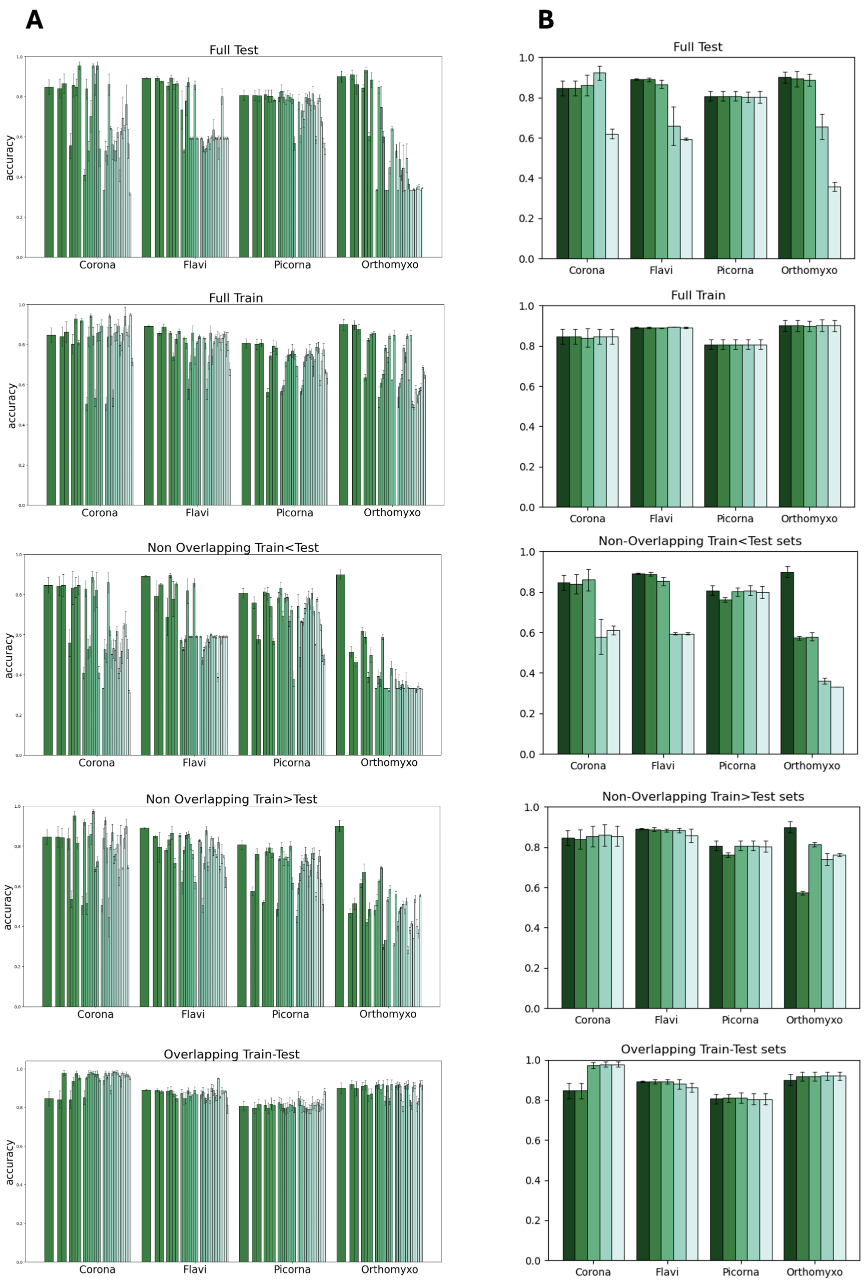


Figure 6: **MENB-H|V accuracies in host prediction from genomic segments in different settings**. **A**: Accuracy over all the segments of genomes at each binary division of sequences until a depth of four (16 segments) in different analysis procedure: Full genomes in the test set, Full genomes in the train set, Non-Overlapping segments with segments in Train $<$ then the ones in the test, Non-Overlapping segments with segments in Train$>$ than the ones in Test, Overlapping Train-Test segments (see Methods 5.3). **B** MaxSum at $\beta = 1$ for the same cases.

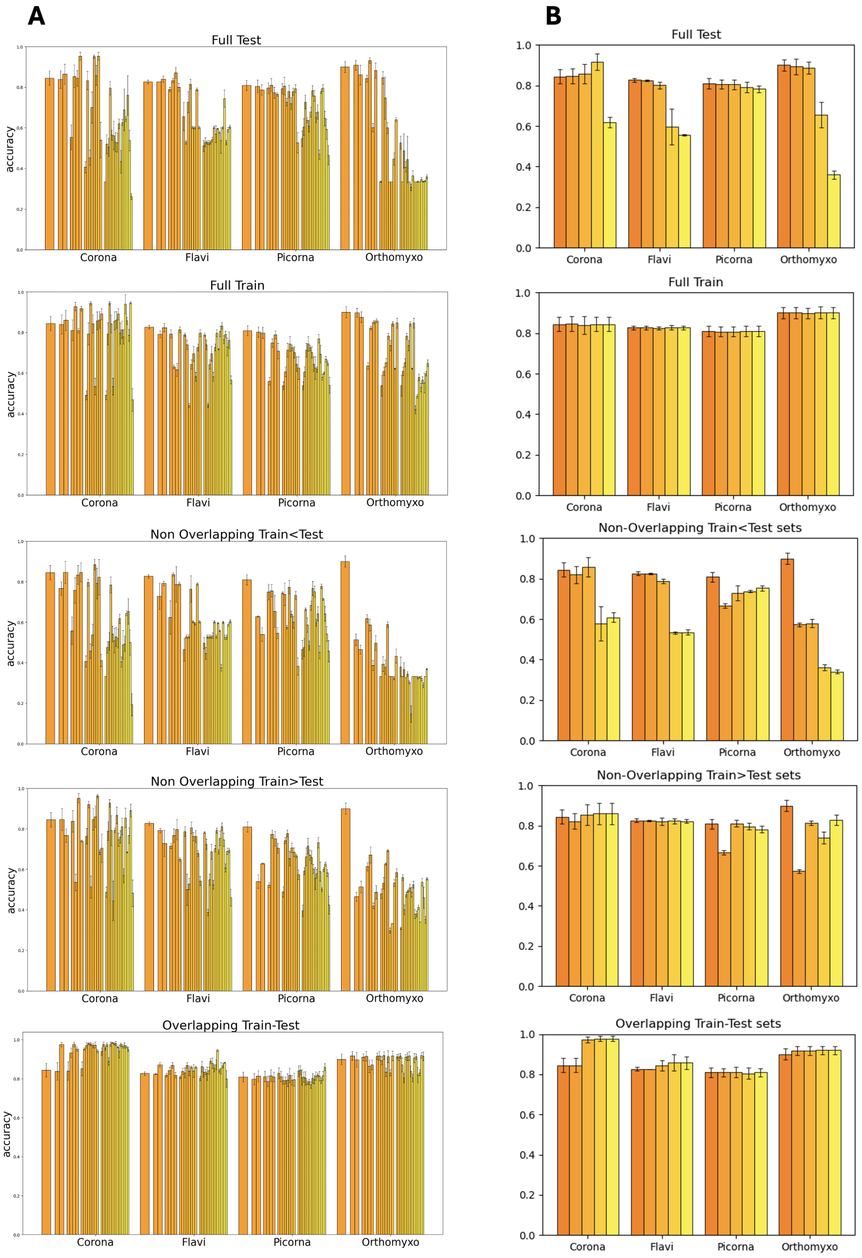


Figure 7: **MENB-H,V accuracies in host prediction from genomic segments in different settings**. **A**: Accuracy over all the segments of genomes at each binary division of sequences until a depth of four (16 segments) in different analysis procedure: Full genomes in the test set, Full genomes in the train set, Non-Overlapping segments with segments in Train < then the ones in the test, Non-Overlapping segments with segments in Train> than the ones in Test, Overlapping Train-Test segments (see Methods 5.3). **B** MaxSum at $\beta = 1$ for the same cases.

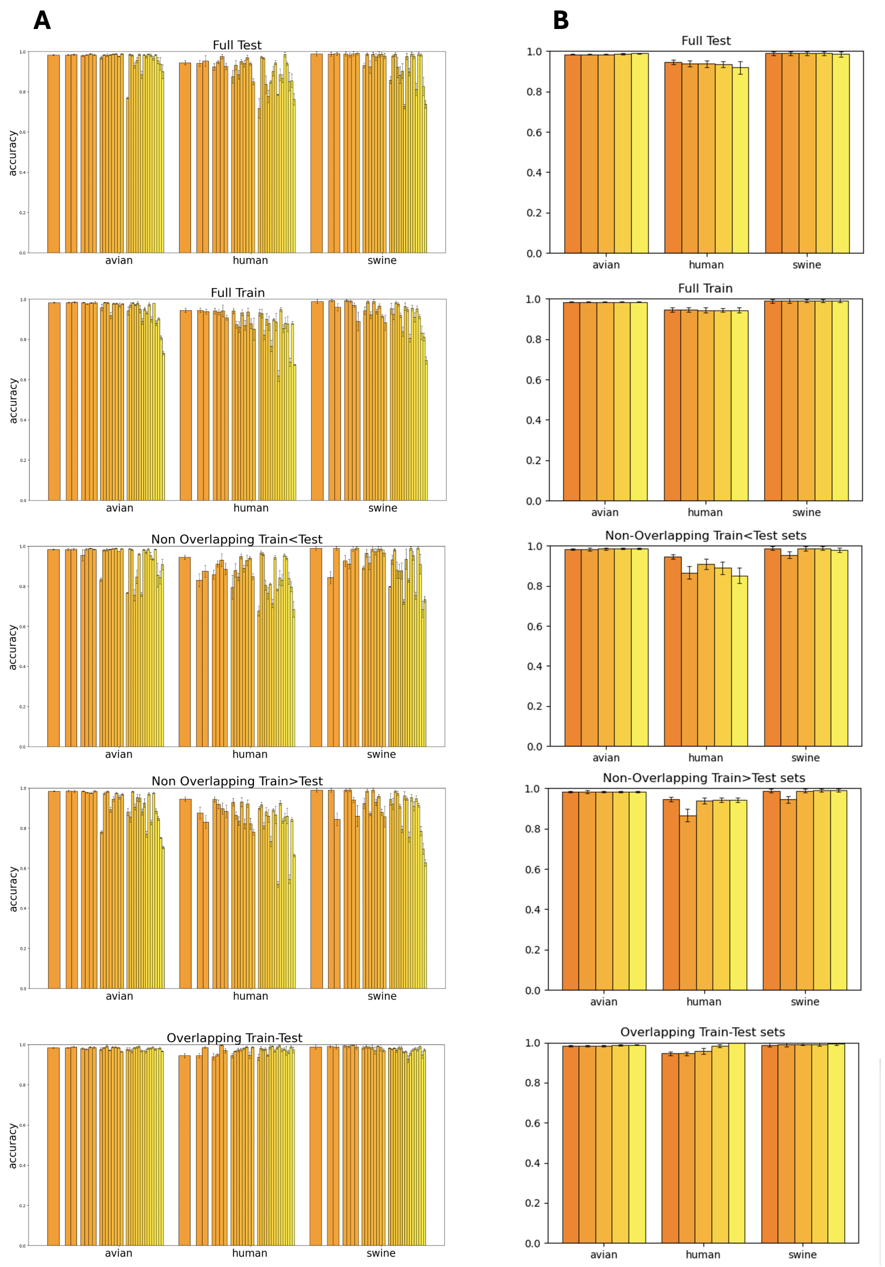


Figure 8: **MENB-H,V accuracies in viral prediction from genomic segments in different settings**. **A**: Accuracy over all the segments of genomes at each binary division of sequences until a depth of four (16 segments) in different analysis procedure: Full genomes in the test set, Full genomes in the train set, Non-Overlapping segments with segments in Train $<$ then the ones in the test, Non-Overlapping segments with segments in Train$>$ than the ones in Test, Overlapping Train-Test segments (see Methods 5.3). **B** MaxSum at $\beta = 1$ for the same cases.

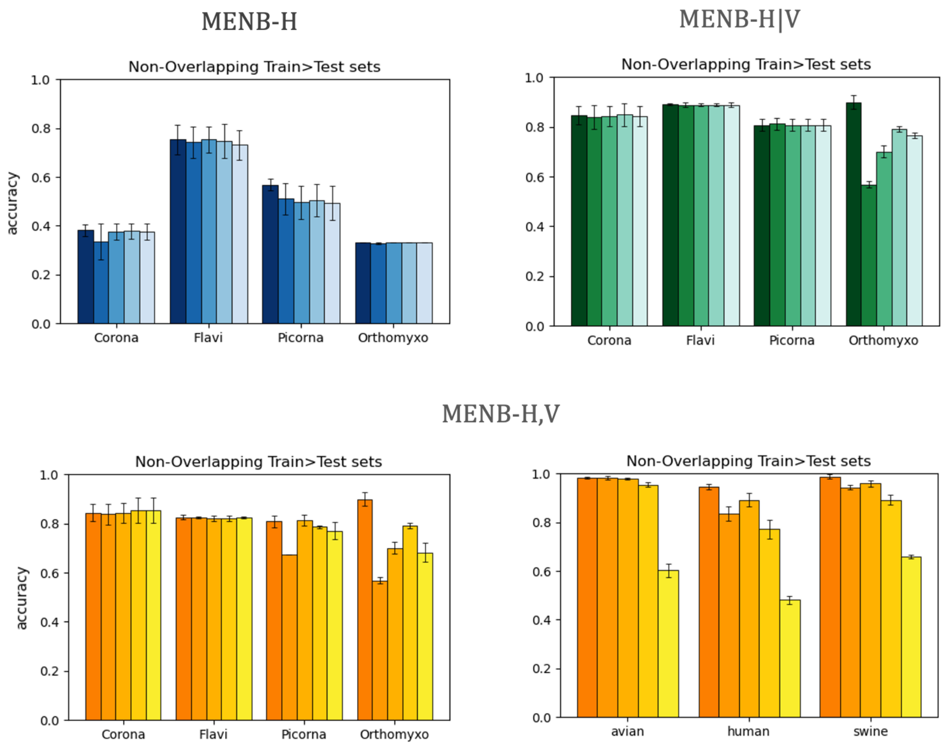


Figure 9: **MaxSum in the Non-Overlapping Train>Test setting**. MaxSum host accuracies at $\beta = 0$ for MENB-H, MENB-H$|V$ (top) and both host and viral prediction accuracies for MENB-H,V (bottom).

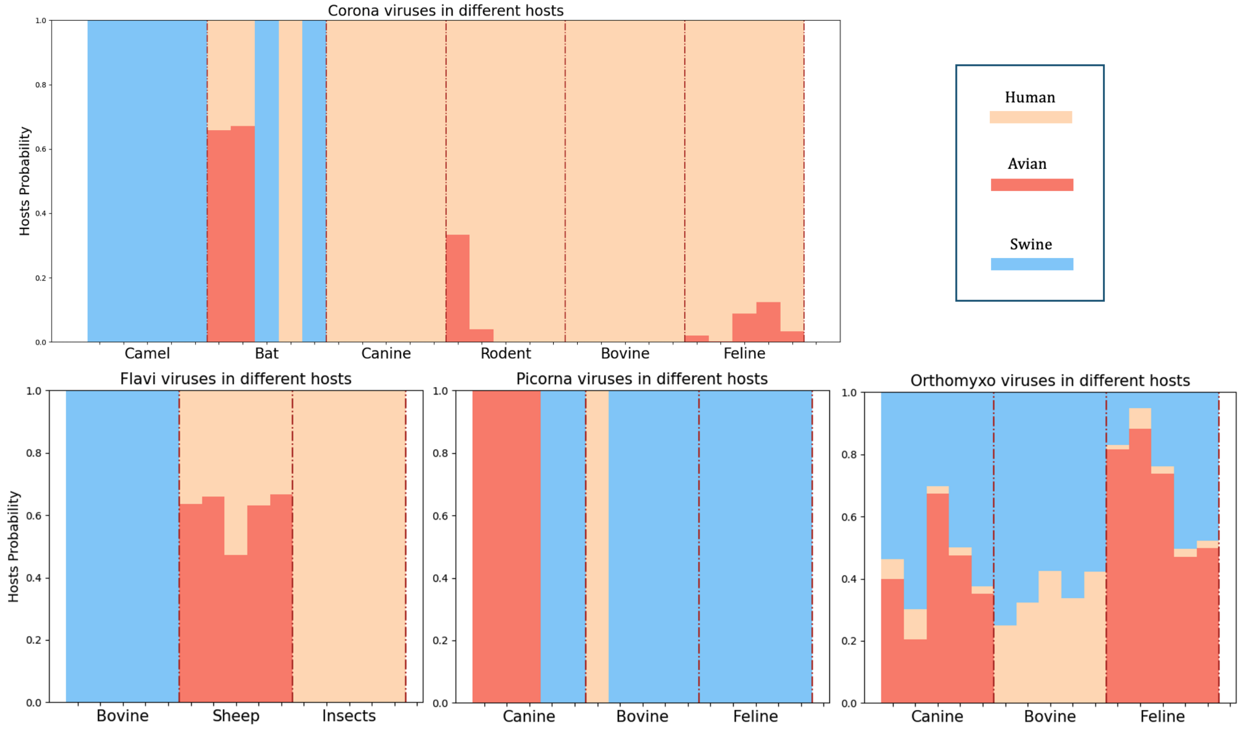


Figure 10: **Average host probabilities from MENB-H,V predictions on a set of sequences belonging to hosts non present in the train data for the four viral families under study**. The viral family is decoded with 100% accuracy on all the sequences considered. For *Orthomyxoviridae*, Canine and Feline sequences are from influenza A genus while bovine is from influenza D genus, a subfamily not included in the train data (see Suppl. Fig. 2D for more details). In particular only PB2 segments were considered for this family, while full genomes in *Picornaviridae*, *Coronaviridae*, and *Flaviviridae* cases shown. The averages are over the MENB-H,V models built on the 3 partitions of the standard train sets (Suppl. Fig. 1).

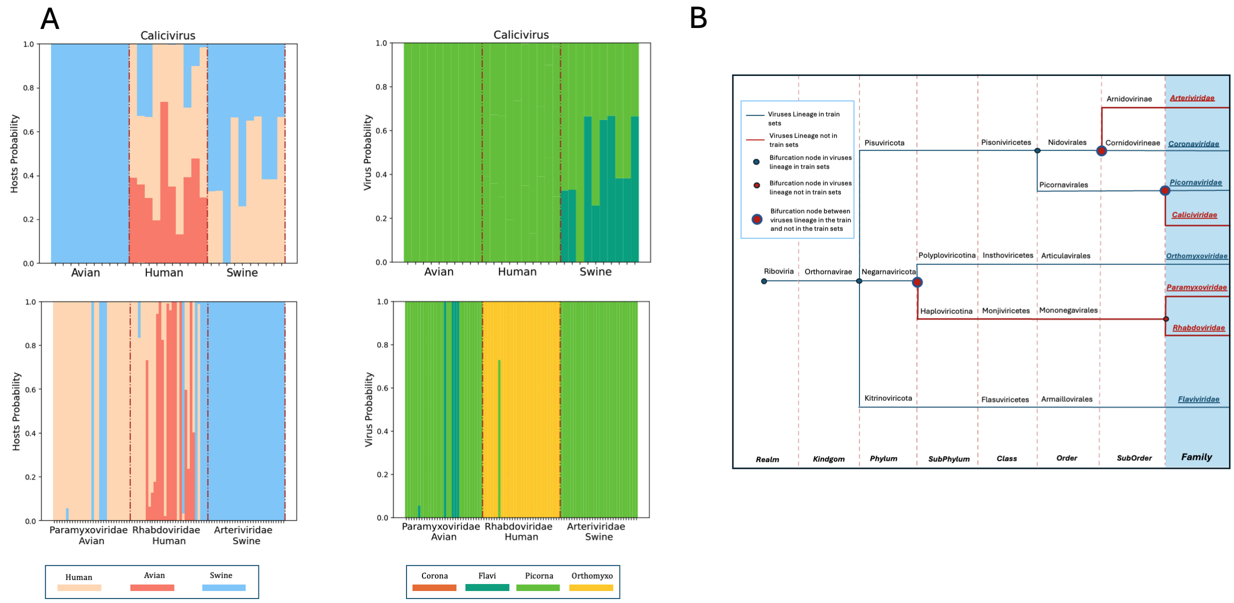


Figure 11: **Average Host and Viral Family probabilities from MENB H,V prediction on sequences of RNA viral families non present in the train-data**. **A**: Host and Viral probabilities assigned by MENB models in Calicivirus (top) and other viral families (bottom). **B**: Taxonomic tree to highlight the connections between all the viral families considered in the paper, from trained ones (shown in blue lines) and non-belonging to train (in red). Calicivirus belong to the order of Picornavirales the same as *Picornaviridae*, Arteriviridae belong to a different suborder from that of *Coronaviridae*, while Paramyxoviridae, Rhabdoviridae are less taxonomically close to the families in the train sets. The averages are over the MENB-H,V models built on the 3 partitions of the standard train sets (Suppl. Fig. 1).

| Non-Overlapping Corona | Accuracy |
|---|---|
| 3'-5' UTR | 0.86 |
| ORF1ab | 1.0 |
| S | 0.97 |
| M | 0.67 |
| N | 0.33 |

Table 3: **Accuracies for genomic regions in *Coronaviridae* and non-overlapping setting.** 90 sequences of *Coronaviridae* for three groups of host, avian, human and swine (see Suppl. Fig. 2B), were used to train models on complementary regions of the ones shown in the table, while testing on the removed portions (of the same sequences).

| Full Train Corona | Accuracy |
|---|---|
| 3'-5' UTR | 0.89 |
| ORF1ab | 1.0 |
| S | 1.0 |
| M | 0.67 |
| N | 0.33 |

Table 4: **MENB H|V accuracy on genomic regions of *Coronaviridae* when training on full length genomes.** The MENB H|V models have been trained on the same set of sequences, taking three sub-samples, and averaging results, to avoid using the same sequences for train and test

| **Segments Length** | ORF1ab | S | M | N | 3'-5' UTR |
|---|---|---|---|---|---|
| Corona Human | 20999 | 4008 | 679 | 1246 | 561 |

Table 5: **Average length for *Coronaviridae* regions**. Average lengths for segments from dataset of 20 *Coronaviridae* sequences (Suppl. Fig. 2A) studied in Suppl. Fig. 12.

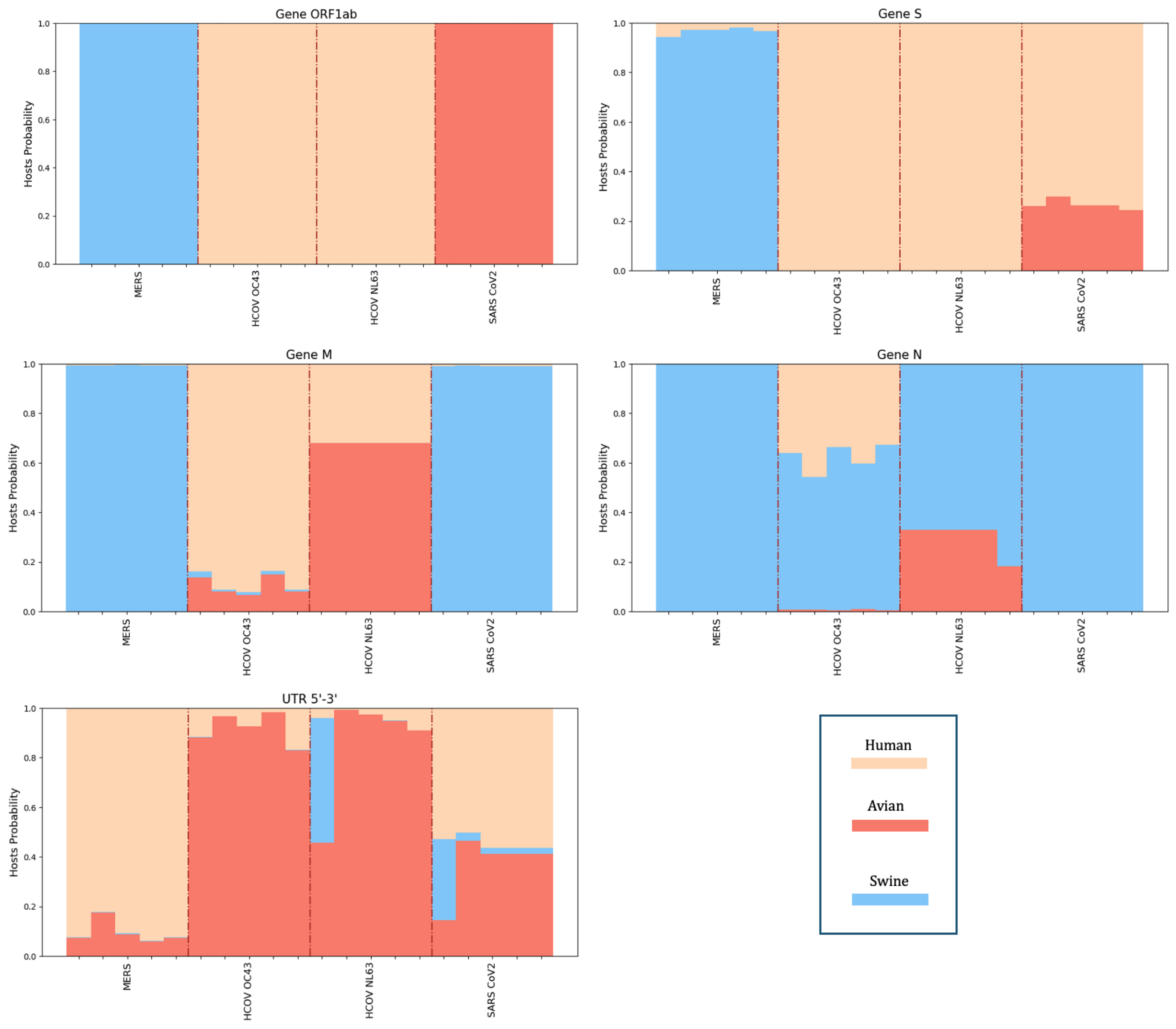


Figure 12: **Average Host Probabilities of MENB-H|V on specific regions of Human *Coronaviridae***. Test of MENB-H|V of ORF1ab, ORFS, ORFM, ORFN, 5'-3' UTR segments in Human sequences of the indicated *Coronaviridae* subfamilies. The averages are over the MENB-H|V models built for *Coronaviridae* on the 3 partitions of the standard train sets (Suppl. Fig. 1).

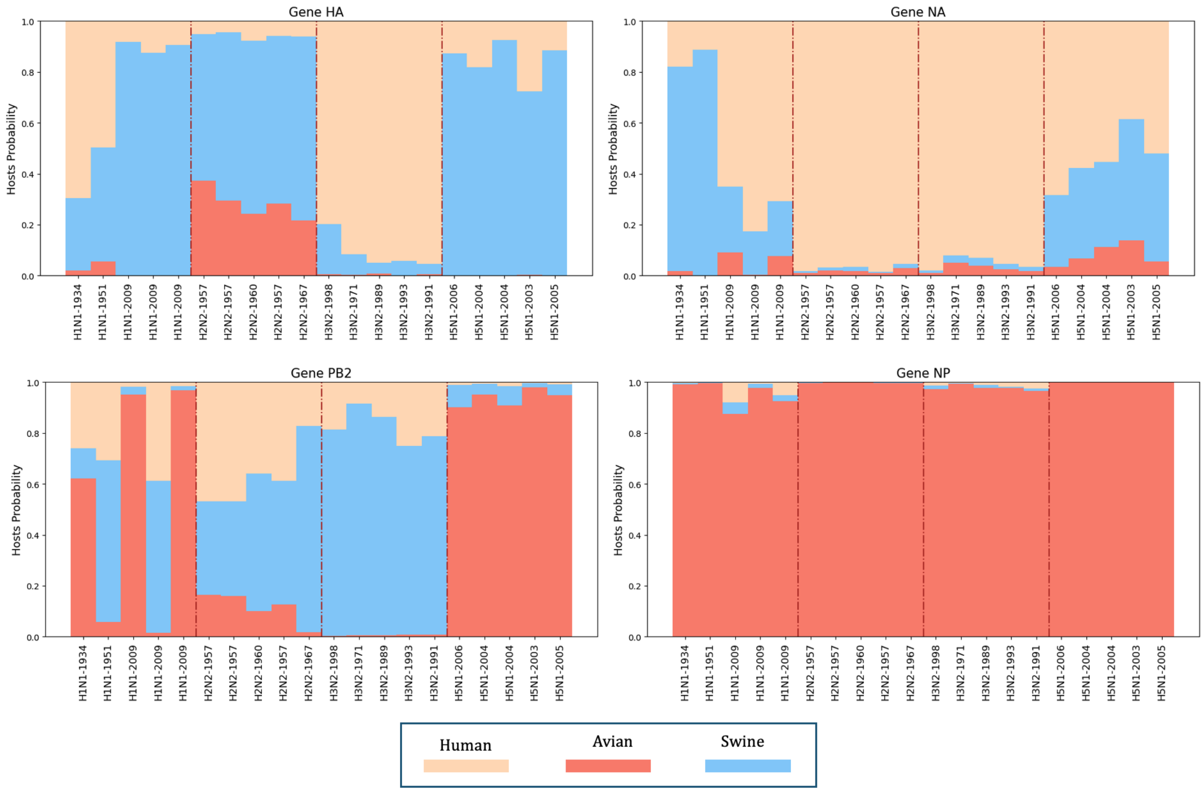


Figure 13: **Average Host Probability of MENB-H|V on specific regions of *Orthomyxoviridae***. MENB-H|V models were used to decode the host on HA, NA, PB2, NP segments in Human sequences of the indicated *Orthomyxoviridae* types. The averages are over the MENB-H|V models built for *Orthomyxoviridae* on the 3 partitions of the standard train set (Suppl. Fig. 1).

| **Segments Length** | HA | NA | PB2 | NP |
|---|---|---|---|---|
| Orthomyxo Human | 1700 | 1393 | 2286 | 1393 |

Table 6: **Average length for *Orthomyxoviridae* regions**. Average lengths for segments from the dataset of 20 *Orthomyxoviridae* sequences (Suppl. Fig. 2A) used in Suppl. Fig. 13

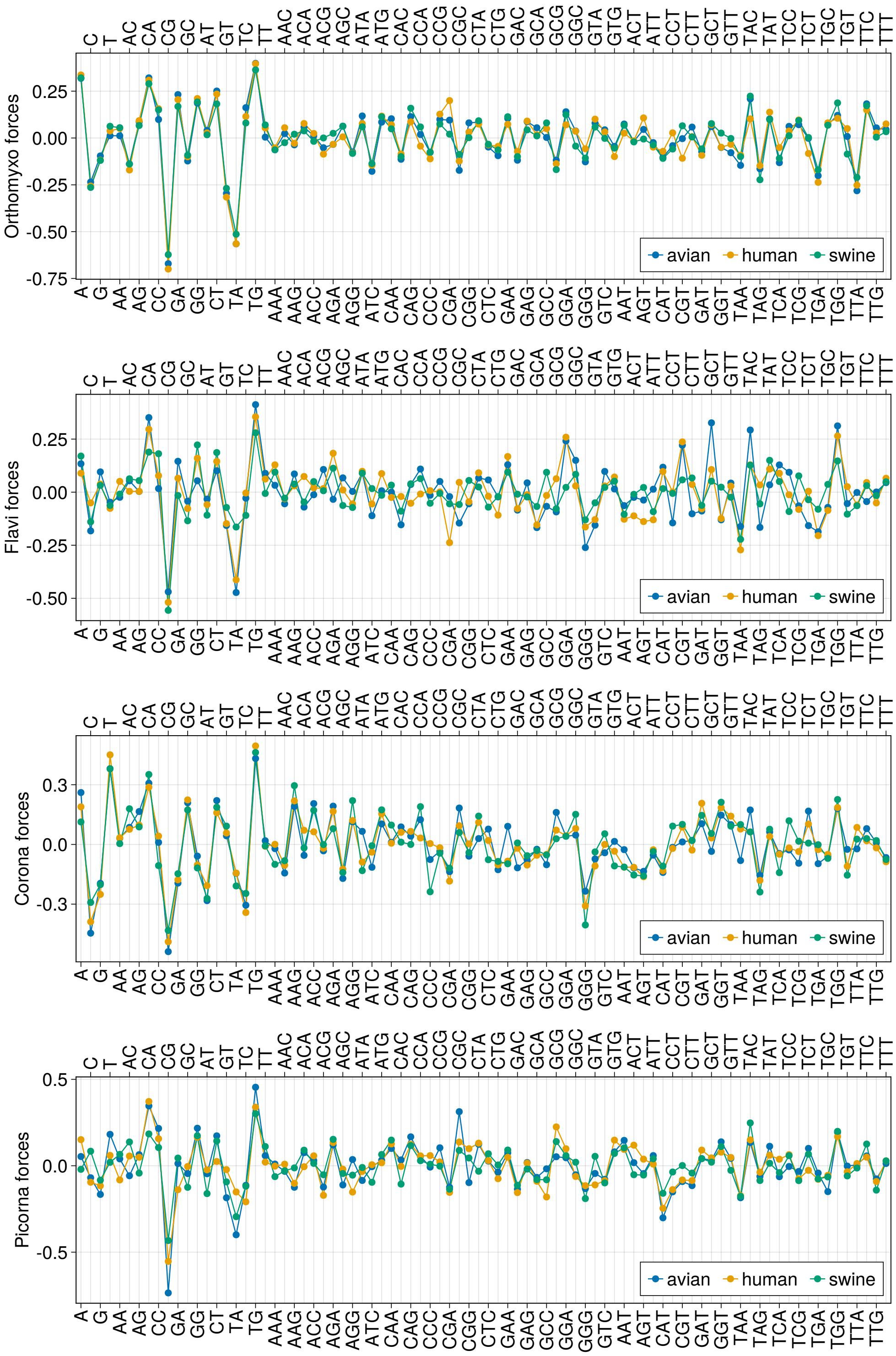


Figure 14: **Force parameters.** All forces shown for each model learned in this work.

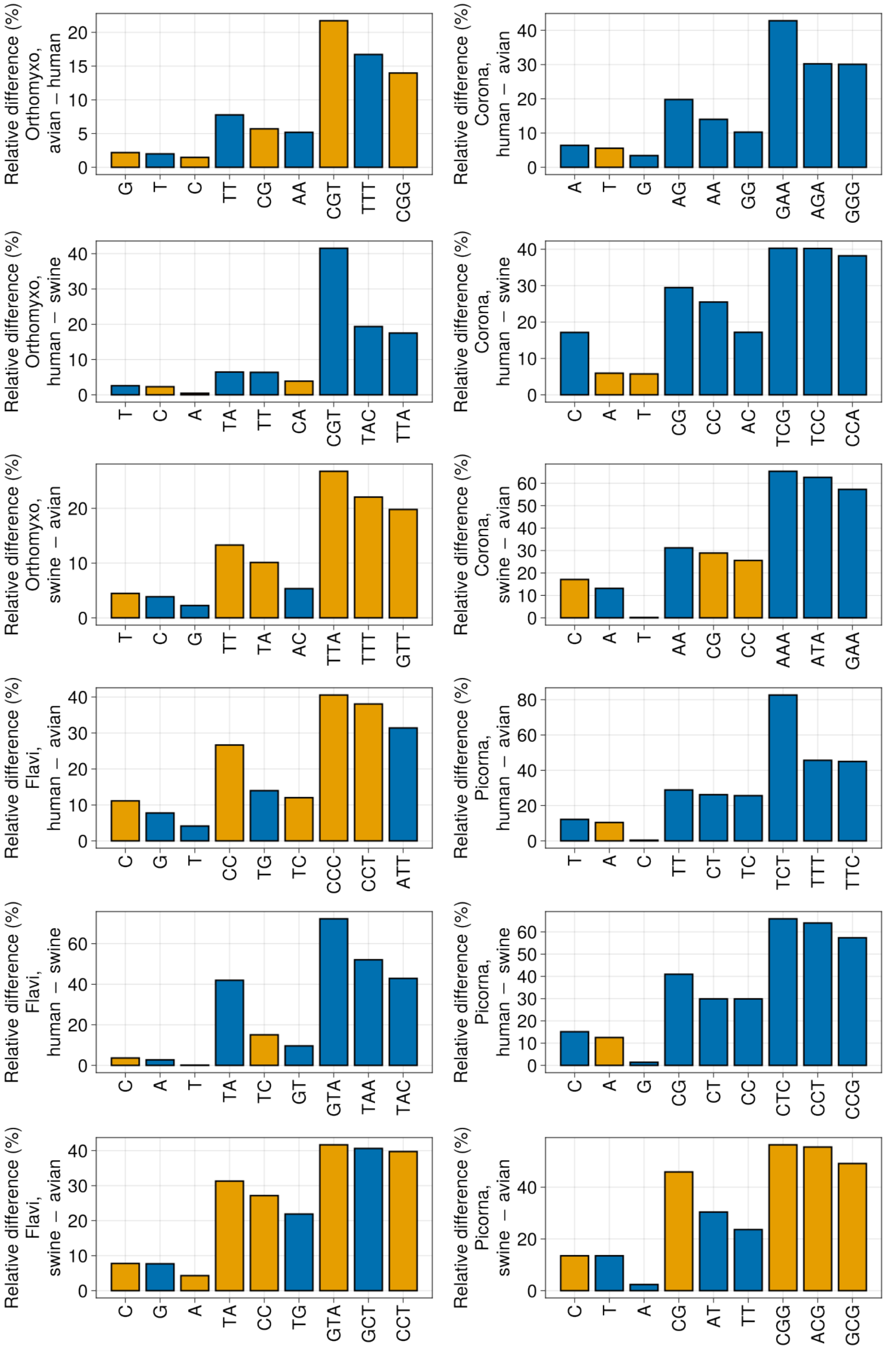


Figure 15: **Relative difference in motif usage shown for each pair of hosts at given viral family**. Blue bars correspond to increases in motif usage, and orange bars to decreases. Only the 3 highest differences (in absolute value) are shown for nucleotides, 2-mers and 3-mers.

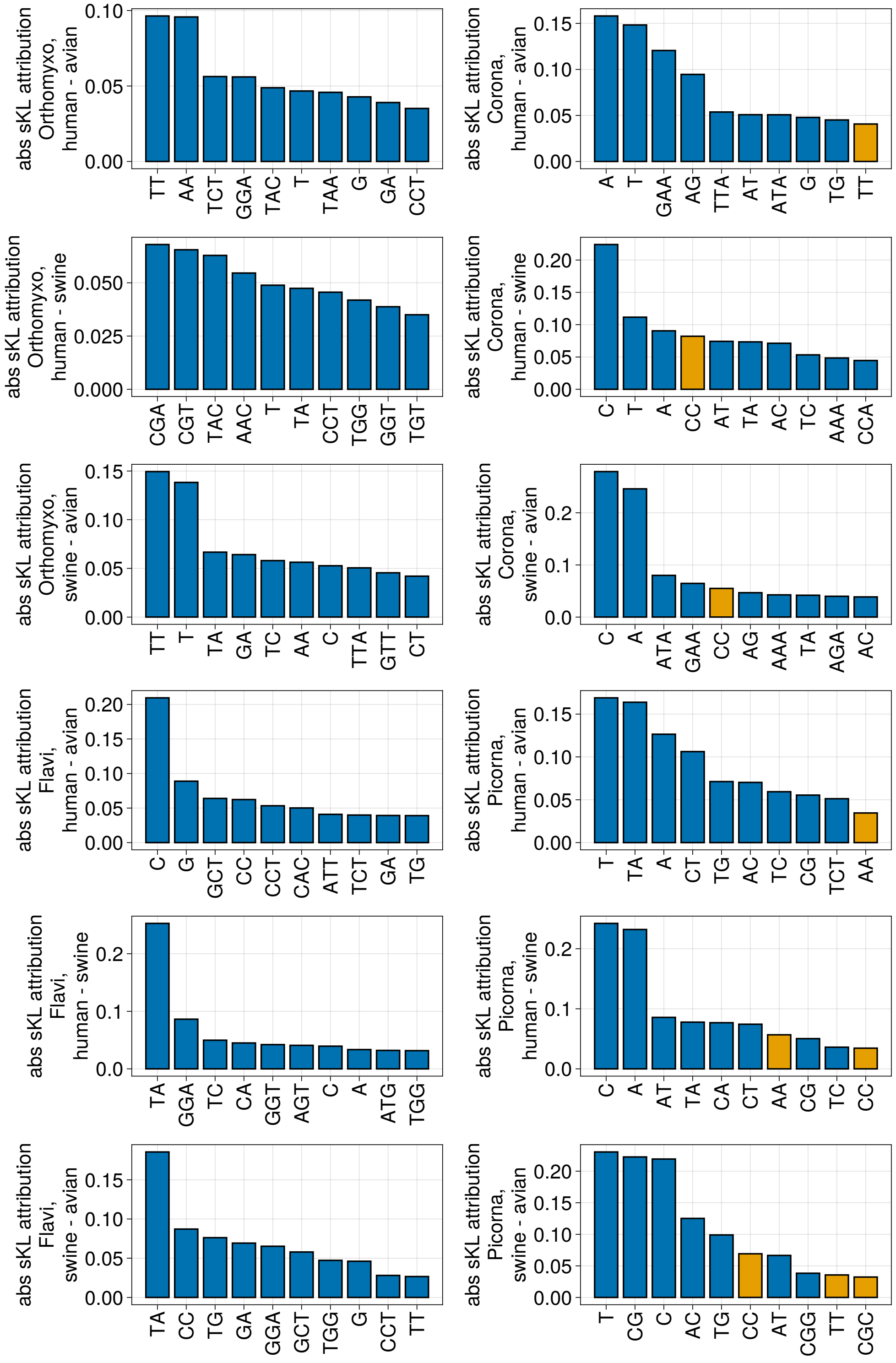


Figure 16: **Attribution to symmetrized KL divergence shown for each pair of hosts at given viral family**. Blue bars correspond to positive attributions, and orange bars to negative attributions. Only the 10 highest attributions (in absolute value) are shown.

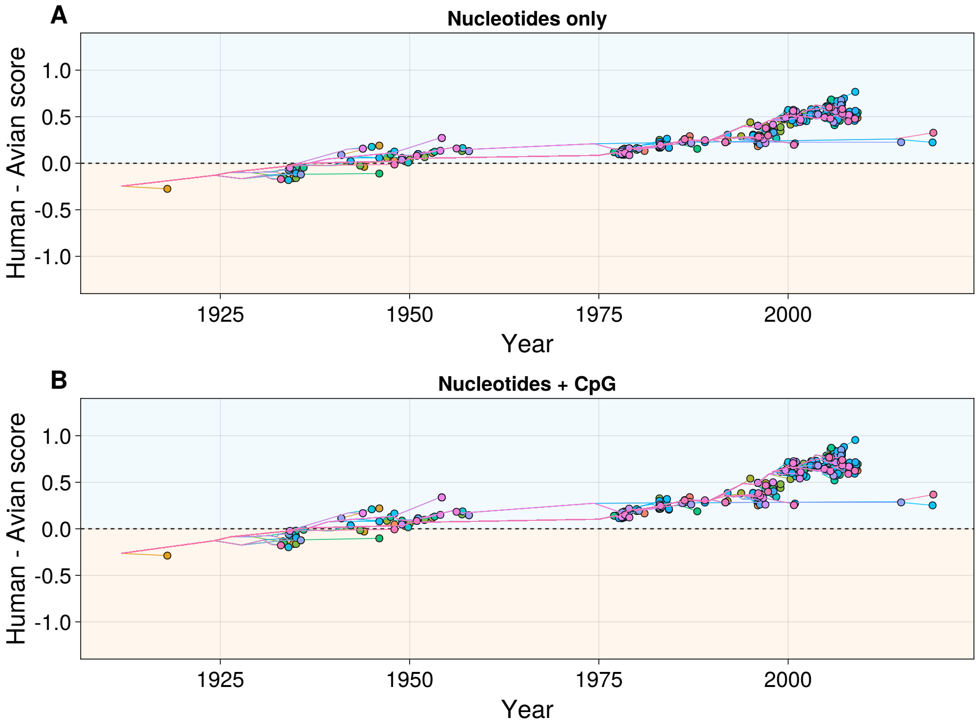


Figure 17: **Log-likelihood differences of simplified MENB *Orthomyxoviridae* human and avian models versus time of H1N1 Influenza A sequences**. In panel **A** a model with only nucleotide force inferred is used, and in panel **B** these forces are inferred together with the CpG force. The colored lines are the reconstructed paths of the inferred phylogenetic tree that connect the root to each leaf (observed sequence), and the score versus inferred time is plotted also for the internal node (inferred) sequences.

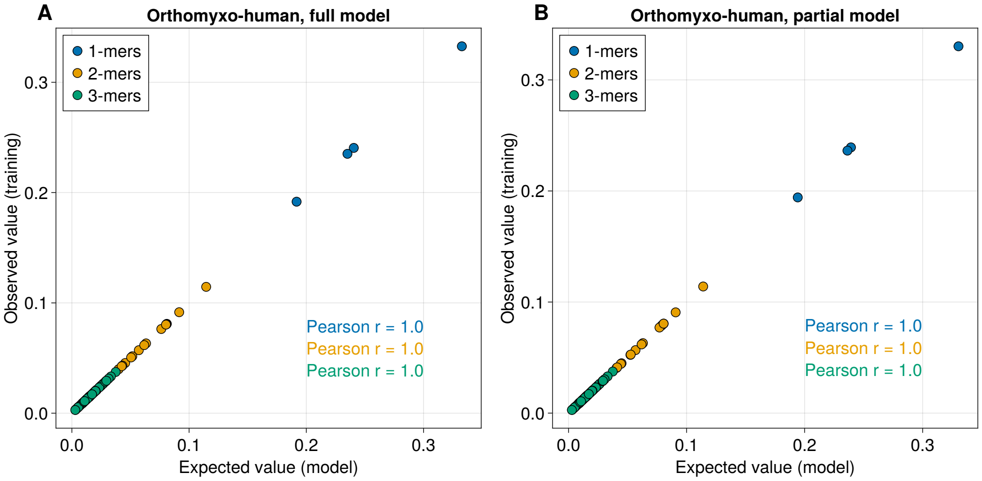


Figure 18: **The model frequencies reproduce the observed ones.** **A**: Frequency of nucleotides, 2-mers and 3-mers observed in the training set of full human *Orthomyxoviridae* sequences versus the value obtained analytically from the inferred MENB model. **B**: Same as **A** for the MENB model trained on human *Orthomyxoviridae* sequences without the segment coding for PB2. .

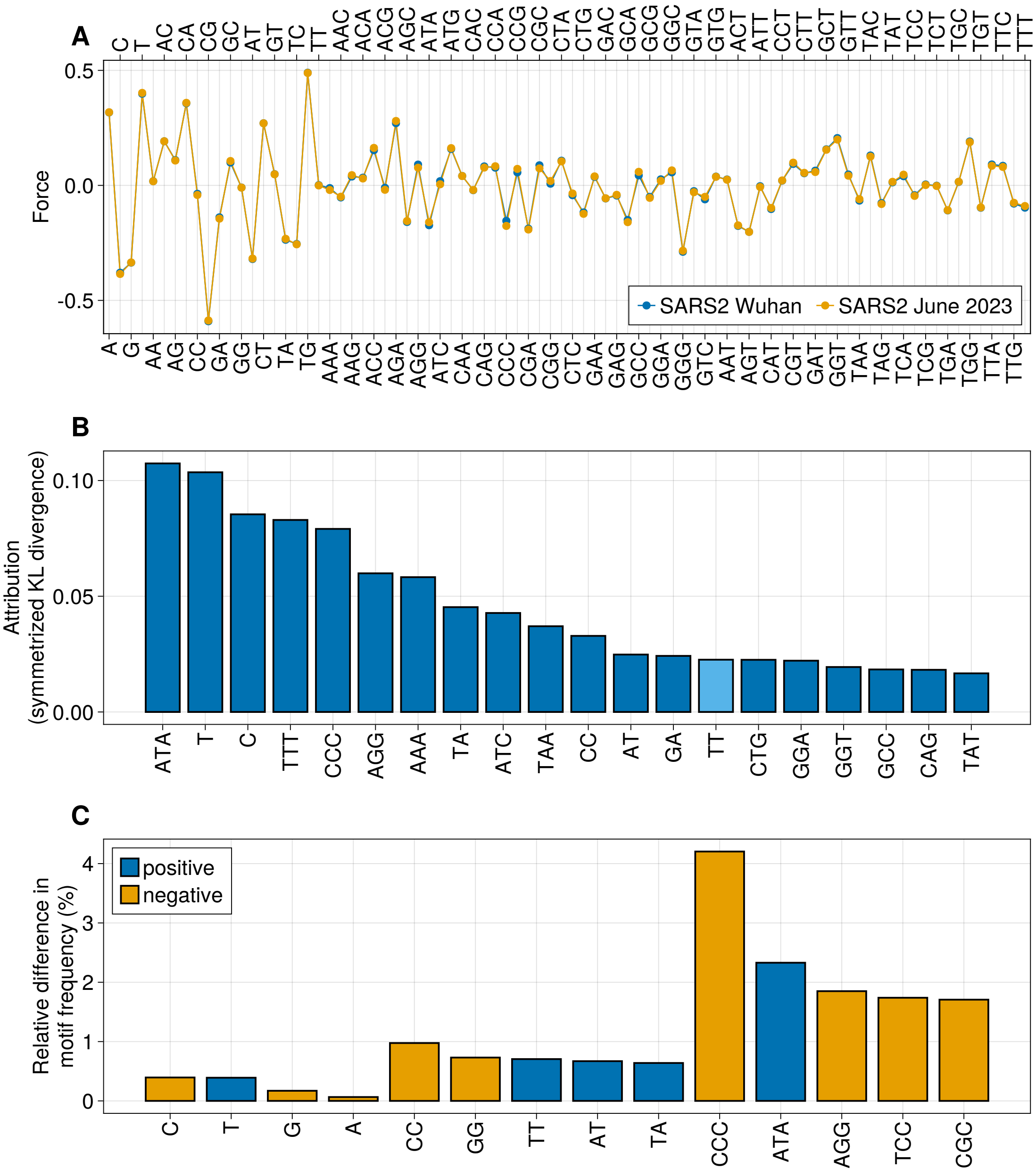


Figure 19: **Analysis of forces changes in SARS-CoV-2.** **A**: Plot of each of the 84 parameters (forces) learned by MENB models trained on the SARS-CoV-2 sequence collected in Wuhan in December 2019 (blue) and on sequences collected in June 2023 (orange). **B**: Attributions computed with the method of integrated gradients (Methods Sec. 5.1.3) for the symmetrized Kullback-Leibler divergence between the MENB models used in panel A. To allow for an easier visualization only the 20 parameters with the highest contribution (in absolute value) to the symmetrized KL divergence are shown. Orange bars denote negative attributions. **C**: Relative difference in expected motif frequencies between the MENB models used in panel **A** (Methods Sec. 5.1.3). Only the 5 top differences (in absolute value) are plotted for 2-mers and 3-mers. Blue (orange) bars correspond to positive (negative) differences.

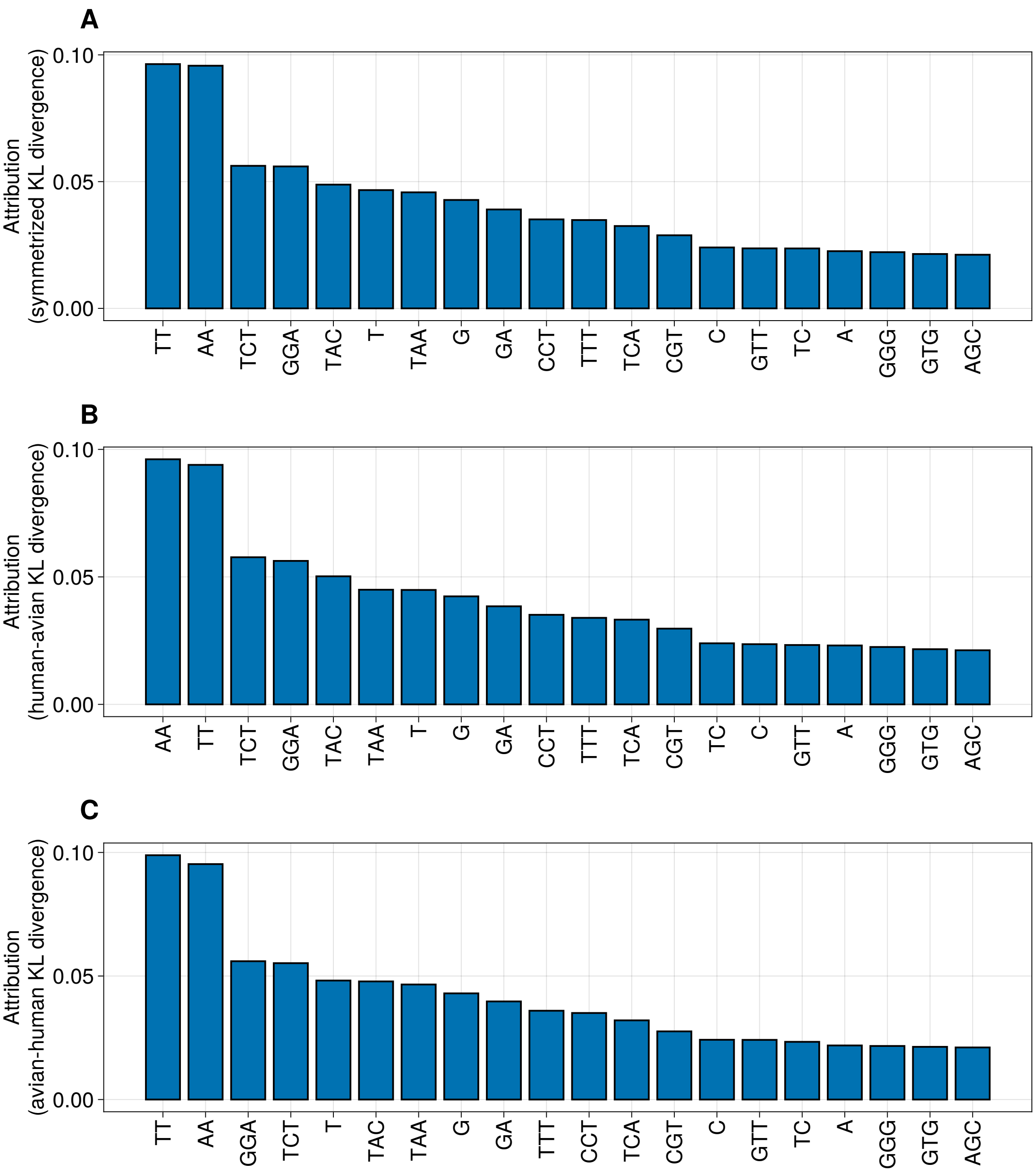


Figure 20: **Comparison of forces between *Orthomyxoviridae* human and avian.** Comparison between attribution to the symmetrized KL divergence between *Orthomyxoviridae* human and avian viruses (panel **A**), and the two non-symmetrized KL divergences that compose it (panels **B**, **C**).

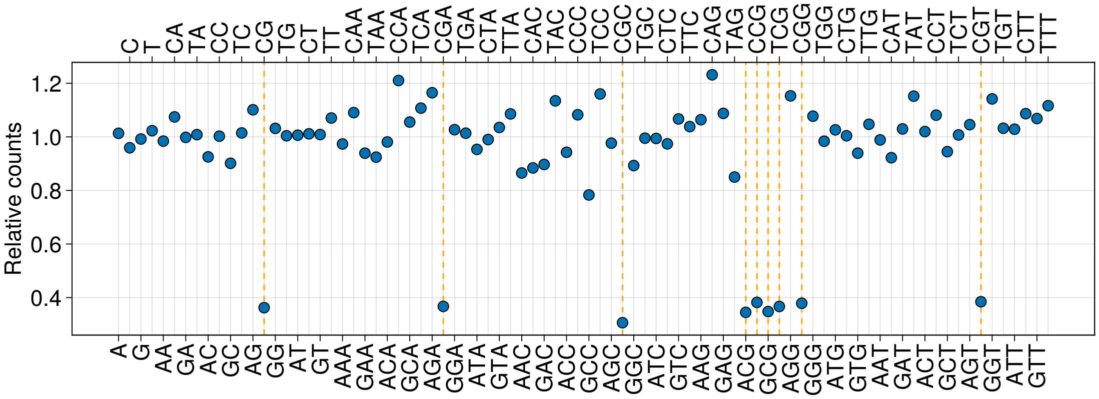


Figure 21: **Comparison between the number of motif observed in the 1918 H1N1 PB2 sequence and in PB2-coding sequence synthetically evolved to reduce their CpG number.** A value of 1 means no change in motif abundance. CpG-containing motifs are highlighted with orange lines.

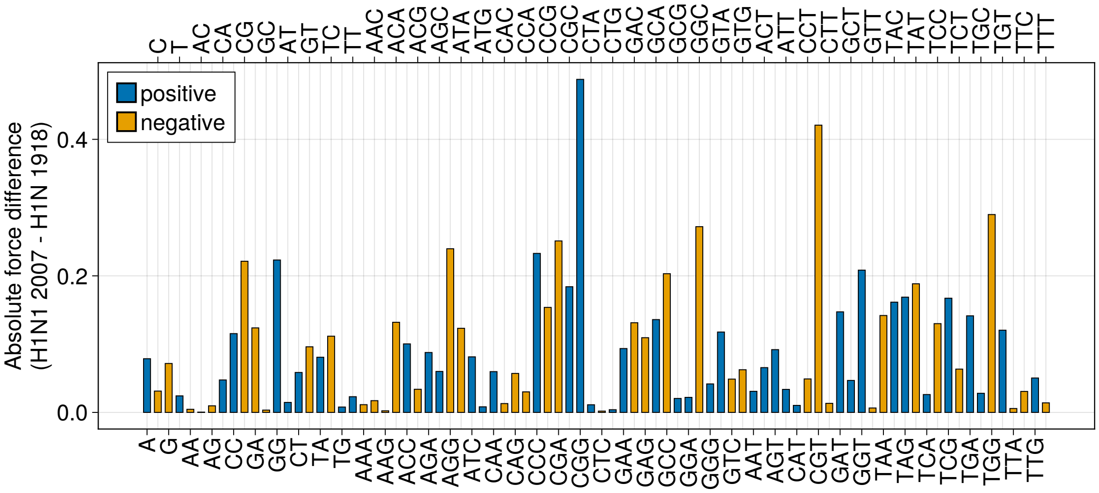


Figure 22: **Comparison between the forces inferred on the 1918 and in 2007 H1N1 sequences.** Blue/orange bars correspond to increased/decreased forces of 2007 sequences with respect to the 1918 sequence.
